# Supplementary material for: Genotype-by-Diet Interactions for Larval Performance and Body Composition Traits in the Black Soldier Fly, Hermetia illucens
Source: Insects. 2022 Apr 30;13(5):424. doi: 10.3390/insects13050424 (PMC9147266; doi:10.3390/insects13050424)

Supplementary Information for

Genotype-by-diet interactions for larval performance and body composition traits in the black soldier fly, *Hermetia illucens*

Christoph Sandrock, Simon Leupi, Jens Wohlfahrt, Cengiz Kaya, Maike Heuel, Melissa Terranova, Wolf U. Blanckenhorn, Wilhelm Windisch, Michael Kreuzer, Florian Leiber.

Corresponding author:

Christoph Sandrock, Research Institute of Organic Agriculture (FiBL), Department of Livestock Sciences, Ackerstrasse 113, 5070 Frick, Switzerland;

Email: christoph.sandrock@fibl.org

Supplementary Materials – Table of contents

Table S1. Summary of genetic, biogeographic and further provenance characteristics of experimental black soldier fly strains. ... 3

Table S2. Pairwise sequence divergence of mitochondrial COI haplotypes detected across experimental black soldier fly strains. ... 3

Table S3. Microsatellite-specific population genetic characteristics across experimental black soldier fly strains. ... 4

Table S4. Larval growth dynamics: pairwise contrasts within main effects (strain, diet and time). ... 4

Table S5. Dynamic larval growth: threefold interaction contrasts across the factors strain, diet and experimental period. ... 5

Table S6. Dynamic larval growth: pairwise contrasts between strains within factorial levels for diet and assessment day. ... 6

Table S7. Twofold interaction contrasts of pairwise strain and diet level comparisons for larval performance and body composition responses. ... 7

Table S8. Pairwise contrasts between strains within dietary levels for larval performance and body composition responses. ... 10

Table S9. Twofold interaction contrasts of pairwise strain and diet level comparisons for larval amino acid profiles (g/100 g protein). ... 13

Table S10. Pairwise contrasts between strains within dietary levels for larval amino acid profiles (g/100 g protein). ... 16

Table S11. Pairwise contrasts between diets within strains for larval amino acid profiles (g/100 g protein). ... 19

Table S12. Specific protein concentration and nitrogen-to-protein conversion: Twofold interaction contrasts of pairwise strain and diet level comparisons, pairwise contrasts between strains within dietary levels, and pairwise contrasts between diets within strains. ... 21

Figure S1. Mitochondrial COI haplotype relatedness among experimental black soldier fly strains. 22
Figure S2. Larval weights at harvest. 22
Figure S3. Larval mortality. 23
Figure S4. Larval biomass production. 23
Figure S5. Larval estimated protein concentrations. 24
Figure S6. Total amounts of larval estimated protein. 24
Figure S7. Larval ether extract concentrations. 25
Figure S8. Total amounts of larval ether extract. 25
Figure S9. Ratios of larval estimated protein to ether extract. 26
Figure S10. Larval total ash concentrations. 26
Figure S11. Amounts of larval total ash. 27
Figure S12. Larval bioconversion efficiency. 27
Figure S13. Larval nitrogen bioconversion efficiency. 28
Figure S14. Reduction of dietary dry matter. 28
Figure S15. Reduction of dietary neutral detergent fibre. 29
Figure S16. Reduction of dietary acid detergent fibre. 29
Figure S17. Reduction of dietary hemicellulose. 30
Figure S18. Systemic dietary dry matter losses (emissions). 30
Figure S19. Larval amino acid profiles. 31
Figure S20. Specific protein concentrations and nitrogen-to-protein conversions. 32

Table S1. Summary of genetic, biogeographic and further provenance characteristics of experimental black soldier fly strains.

Nuclear genetic cluster assignments (based on 15 microsatellite markers) and exemplary mitochondrial haplotype profiling (based on the partial cytochrome c oxidase I (COI) gene) of experimental strains (S1-4) refer to data bases and GenBank Accession numbers provided by Kaya *et al.* (2021) and Ståhls *et al.* (2020), respectively, see reference list included in the main text.

Strain	Nuclear genetic cluster assignment	Mitochondrial Haplotype (COI)	Inferred biogeographic origin & further provenance characteristics	Effective source
S1	Cluster 2	LR792261, LR792262	North America, captive only ('domesticated'): widespread across Europe & Africa	Europe (Switzerland), managed population
S2	Cluster 6	LR792223	Asia, naturalised: regional wild & captive populations	Asia (Malaysia), managed population
S3	Cluster 8	LR792223	Eastern Australia, naturalised: Regional wild & captive populations	Australia (Victoria), managed population
S4	Cluster 1	LR792267	North America, captive only ('domesticated'): widespread across North America, Africa & Europe	Africa (Tunisia), managed population

Table S2. Pairwise sequence divergence of mitochondrial COI haplotypes detected across experimental black soldier fly strains.

Among the four experimental strains (S1-4), S1 harboured two distantly related haplotypes (Ht-I and Ht-II, matching e.g. GenBank accession numbers LR792261 and LR792262) at about equal frequencies, strain 4 harboured a different haplotype exclusively (matching e.g. LR792267), and strains 2 and 3 were both fixed for the same distinct haplotype (matching e.g. LR792223). For more details, see Ståhls *et al.* (2020) in the reference list of the main text. Computations are based on uncorrected p-distances across 658 base pairs of the partial cytochrome c oxidase I (COI) gene. See Figure S1 for visualisation.

	**S1-Ht-I**	**S1-Ht-II**	**S2**	**S3**
**S1-Ht-I**	-			
**S1-Ht-II**	0.033	-		
**S2**	0.038	0.020	-	
**S3**	0.038	0.020	0.000	-
**S4**	0.002	0.035	0.039	0.039

Table S3. Microsatellite-specific population genetic characteristics across experimental black soldier fly strains.

For more details on microsatellite loci, see Kaya *et al.* (2021) as part of the reference list included in the main text. N_A: number of alleles across and within strains (S1-4), respectively (including strain-specific private alleles in parentheses), F_{ST}: fixation index; F_{IS}: inbreeding coefficient; H_{obs}/H_{exp}: observed and expected heterozygosity. Significant *F*-statistics (adjusted for 15 simultaneous tests) are highlighted in bold. Genetic differentiation (F_{ST}) was significant for all loci across strains, yet with notable locus-specific variation. Across strains, F_{IS} was positive for most loci as would be expected, yet significant homozygote excess was detected for two loci only. Significant homozygote excess across loci within strains S1 and S2 (see Table 2) was effectively driven by single loci each, i.e. Hi_3-1 and Hi_1-2, respectively.

Locus	N_A	Strain-specific N_A (plus private N_A)				F_{ST}	F_{IS}	H_{obs}	H_{exp}
		S1	S2	S3	S4				
Hi_1-1	16	5 (1)	10 (6)	6 (2)	6	**0.206**	0.015	0.700	0.711
Hi_1-2	10	4 (1)	5 (1)	7 (4)	3	**0.373**	0.066	0.480	0.514
Hi_1-3	4	4	2	2	4	**0.176**	0.003	0.385	0.386
Hi_1-4	7	4 (1)	4	4 (1)	4 (1)	**0.137**	-0.038	0.650	0.626
Hi_1-5	6	4	2	3 (1)	4	**0.330**	0.084	0.500	0.546
Hi_2-1	6	2	4	6 (2)	1	**0.172**	0.027	0.400	0.411
Hi_2-2	11	5	6 (2)	7 (2)	5	**0.140**	**0.129**	0.655	0.752
Hi_2-3	8	3 (1)	4	5 (1)	2	**0.361**	0.094	0.515	0.568
Hi_2-4	7	4	3	4 (1)	4	**0.256**	0.102	0.575	0.641
Hi_2-5	4	3	2 (1)	1	3	**0.383**	-0.042	0.330	0.317
Hi_3-1	9	6 (3)	2 (1)	2	5 (2)	**0.499**	**0.333**	0.205	0.307
Hi_3-2	7	4 (1)	4 (1)	4 (1)	3	**0.231**	-0.040	0.605	0.582
Hi_3-3	8	5	4 (1)	4 (1)	5	**0.213**	0.093	0.520	0.573
Hi_3-4	8	3	2	6 (3)	4	**0.324**	0.106	0.465	0.520
Hi_3-5	8	4 (1)	4	6 (1)	3	**0.292**	0.128	0.540	0.619
Overall	*118*	*60 (9)*	*58 (13)*	*67 (20)*	*55 (3)*	***0.273***	***0.068***	*0.502*	*0.538*

Table S4. Larval growth dynamics: pairwise contrasts within main effects (strain, diet and time).

Estimates of differences or changes in live weight (mg) are reported on the response scale. Only comparisons between successive assessment dates are shown. Note that given the significant interactions among all three main effects, these pairwise contrasts between levels within a given factor across levels of the other factors must be interpreted with caution. See Tables S5 and S6 for in-depth inspections of formally correct interaction contrasts.

Contrast	Estimate	*P* value
Strain: S2 – S1	-15.47	< 0.001
Strain: S3 – S1	-0.93	0.999
Strain: S4 – S1	-3.58	0.155
Strain: S3 – S2	14.54	< 0.001
Strain: S4 – S2	11.89	< 0.001
Strain: S4 – S3	-2.66	0.524
Diet: FW – PF	-18.98	< 0.001
Diet: PM – PF	-57.35	< 0.001
Diet: PM - FW	-38.38	< 0.001
Day: 4 - 0	33.94	< 0.001
Day: 8 - 4	101.81	< 0.001
Day: 12 - 8	50.55	< 0.001
Day: 15 - 12	-4.07	0.060

Table S5. Dynamic larval growth: threefold interaction contrasts across the factors strain, diet and experimental period.

Simultaneous testing was applied and pairwise level combinations are indicated for each of the three factors strain (S1-4), diet (poultry feed (PF), food waste (FW), poultry manure (PM)) and period (assessment days between 0 (start) and harvest). Only periods with applied relevance, i.e. successive assessment days, or entire periods until day 12 or harvest are reported. Value refers to contrasting across pairwise contrasts for all factors (analogous to estimates), and per definition each interaction contrast has one degrees of freedom. *P*-values are adjusted for multiple testing.

Strain	Diet	Period	Value	χ^2	*P*	
S1-S2	PF-FW	0-4	-20.52	17.76	0.003	**
S1-S3	PF-FW	0-4	-19.34	15.77	0.008	**
S1-S4	PF-FW	0-4	-21.83	20.10	0.001	***
S2-S3	PF-FW	0-4	1.18	0.06	1.000	
S2-S4	PF-FW	0-4	-1.31	0.07	1.000	
S3-S4	PF-FW	0-4	-2.49	0.26	1.000	
S1-S2	PF-PM	0-4	-7.85	2.60	1.000	
S1-S3	PF-PM	0-4	-9.66	3.94	1.000	
S1-S4	PF-PM	0-4	-8.56	3.09	1.000	
S2-S3	PF-PM	0-4	-1.81	0.14	1.000	
S2-S4	PF-PM	0-4	-0.71	0.02	1.000	
S3-S4	PF-PM	0-4	1.10	0.05	1.000	
S1-S2	FW-PM	0-4	12.67	6.77	0.731	
S1-S3	FW-PM	0-4	9.68	3.95	1.000	
S1-S4	FW-PM	0-4	13.27	7.43	0.525	
S2-S3	FW-PM	0-4	-2.99	0.38	1.000	
S2-S4	FW-PM	0-4	0.60	0.02	1.000	
S3-S4	FW-PM	0-4	3.59	0.55	1.000	
S1-S2	PF-FW	4-8	-13.50	7.69	0.467	
S1-S3	PF-FW	4-8	-22.28	20.93	0.001	***
S1-S4	PF-FW	4-8	0.61	0.02	1.000	
S2-S3	PF-FW	4-8	-8.78	3.25	1.000	
S2-S4	PF-FW	4-8	14.11	8.40	0.334	
S3-S4	PF-FW	4-8	22.89	22.10	<0.001	***
S1-S2	PF-PM	4-8	-18.67	14.70	0.013	*
S1-S3	PF-PM	4-8	5.28	1.17	1.000	
S1-S4	PF-PM	4-8	1.44	0.09	1.000	
S2-S3	PF-PM	4-8	23.94	24.18	<0.001	***
S2-S4	PF-PM	4-8	20.11	17.06	0.004	**
S3-S4	PF-PM	4-8	-3.83	0.62	1.000	
S1-S2	FW-PM	4-8	-5.17	1.13	1.000	
S1-S3	FW-PM	4-8	27.56	32.03	<0.001	***
S1-S4	FW-PM	4-8	0.83	0.03	1.000	
S2-S3	FW-PM	4-8	32.72	45.16	<0.001	***
S2-S4	FW-PM	4-8	6.00	1.52	1.000	
S3-S4	FW-PM	4-8	-26.72	30.12	<0.001	***
S1-S2	PF-FW	8-12	12.33	6.42	0.859	
S1-S3	PF-FW	8-12	6.00	1.52	1.000	
S1-S4	PF-FW	8-12	-18.83	14.96	0.012	*
S2-S3	PF-FW	8-12	-6.33	1.69	1.000	
S2-S4	PF-FW	8-12	-31.17	40.97	<0.001	***
S3-S4	PF-FW	8-12	-24.83	26.01	<0.001	***
S1-S2	PF-PM	8-12	-28.72	34.80	<0.001	***
S1-S3	PF-PM	8-12	-29.61	36.99	<0.001	***
S1-S4	PF-PM	8-12	-26.11	28.76	<0.001	***
S2-S3	PF-PM	8-12	-0.89	0.03	1.000	
S2-S4	PF-PM	8-12	2.61	0.29	1.000	
S3-S4	PF-PM	8-12	3.50	0.52	1.000	
S1-S2	FW-PM	8-12	-41.06	71.10	<0.001	***
S1-S3	FW-PM	8-12	-35.61	53.49	<0.001	***
S1-S4	FW-PM	8-12	-7.28	2.23	1.000	
S2-S3	FW-PM	8-12	5.44	1.25	1.000	
S2-S4	FW-PM	8-12	33.78	48.13	<0.001	***
S3-S4	FW-PM	8-12	28.33	33.86	<0.001	***

Strain	Diet	Period	Value	χ^2	*P*	
S1-S2	PF-FW	12-harvest	7.65	2.47	1.000	
S1-S3	PF-FW	12-harvest	-5.88	1.46	1.000	
S1-S4	PF-FW	12-harvest	5.62	1.33	1.000	
S2-S3	PF-FW	12-harvest	-13.52	7.72	0.465	
S2-S4	PF-FW	12-harvest	-2.03	0.17	1.000	
S3-S4	PF-FW	12-harvest	11.50	5.58	1.000	
S1-S2	PF-PM	12-harvest	14.46	8.82	0.272	
S1-S3	PF-PM	12-harvest	2.74	0.32	1.000	
S1-S4	PF-PM	12-harvest	-2.65	0.30	1.000	
S2-S3	PF-PM	12-harvest	-11.71	5.79	1.000	
S2-S4	PF-PM	12-harvest	-17.11	12.35	0.045	*
S3-S4	PF-PM	12-harvest	-5.40	1.23	1.000	
S1-S2	FW-PM	12-harvest	6.81	1.96	1.000	
S1-S3	FW-PM	12-harvest	8.62	3.13	1.000	
S1-S4	FW-PM	12-harvest	-8.27	2.89	1.000	
S2-S3	FW-PM	12-harvest	1.81	0.14	1.000	
S2-S4	FW-PM	12-harvest	-15.08	9.59	0.183	
S3-S4	FW-PM	12-harvest	-16.89	12.04	0.052	.
S1-S2	PF-FW	0-12	-21.69	19.84	0.001	**
S1-S3	PF-FW	0-12	-35.62	53.51	<0.001	***
S1-S4	PF-FW	0-12	-40.05	67.66	<0.001	***
S2-S3	PF-FW	0-12	-13.93	8.18	0.363	
S2-S4	PF-FW	0-12	-18.36	14.23	0.017	*
S3-S4	PF-FW	0-12	-4.43	0.83	1.000	
S1-S2	PF-PM	0-12	-55.24	128.70	<0.001	***
S1-S3	PF-PM	0-12	-33.99	48.74	<0.001	***
S1-S4	PF-PM	0-12	-33.22	46.55	<0.001	***
S2-S3	PF-PM	0-12	21.24	19.04	0.002	**
S2-S4	PF-PM	0-12	22.01	20.44	0.001	***
S3-S4	PF-PM	0-12	0.77	0.03	1.000	
S1-S2	FW-PM	0-12	-33.55	47.48	<0.001	***
S1-S3	FW-PM	0-12	1.62	0.11	1.000	
S1-S4	FW-PM	0-12	6.83	1.97	1.000	
S2-S3	FW-PM	0-12	35.17	52.18	<0.001	***
S2-S4	FW-PM	0-12	40.38	68.77	<0.001	***
S3-S4	FW-PM	0-12	5.21	1.14	1.000	
S1-S2	PF-FW	0-harvest	-14.04	8.31	0.342	
S1-S3	PF-FW	0-harvest	-41.49	72.62	<0.001	***
S1-S4	PF-FW	0-harvest	-34.43	50.01	<0.001	***
S2-S3	PF-FW	0-harvest	-27.45	31.79	<0.001	***
S2-S4	PF-FW	0-harvest	-20.39	17.54	0.003	**
S3-S4	PF-FW	0-harvest	7.06	2.10	1.000	
S1-S2	PF-PM	0-harvest	-40.78	70.15	<0.001	***
S1-S3	PF-PM	0-harvest	-31.25	41.19	<0.001	***
S1-S4	PF-PM	0-harvest	-35.87	54.29	<0.001	***
S2-S3	PF-PM	0-harvest	9.53	3.83	1.000	
S2-S4	PF-PM	0-harvest	4.91	1.01	1.000	
S3-S4	PF-PM	0-harvest	-4.63	0.90	1.000	
S1-S2	FW-PM	0-harvest	-26.74	30.16	<0.001	***
S1-S3	FW-PM	0-harvest	10.25	4.43	1.000	
S1-S4	FW-PM	0-harvest	-1.44	0.09	1.000	
S2-S3	FW-PM	0-harvest	36.99	57.70	<0.001	***
S2-S4	FW-PM	0-harvest	25.30	26.99	<0.001	***
S3-S4	FW-PM	0-harvest	-11.69	5.76	1.000	

Table S6. Dynamic larval growth: pairwise contrasts between strains within factorial levels for diet and assessment day.

Simultaneous testing of strain-specific (S1-4) contrasts within dietary levels (PF: poultry feed; FW: food waste; PM: poultry manure) on individual assessment days (subtables a-d corresponding to day 0, 4, 8 and 12, see Table S8a for evaluations of larval weights at harvest). Estimates are given on the response scale and *P*-values were adjusted for multiple testing.

Briefly summarised, no differences were detected for initial larval weights between strains on any diet. Until day 4, S1 grew faster than S2 and S3 on PF, but remained smaller than all other strains on FW, where larvae of S4 larvae were the heaviest. On PM, larvae of S2 were lighter compared to S4 on day 4. By day 8 all strains differed significantly from each other on PF, except for the comparison between S3 and S4. Similarly, except for the comparison between S1 and S4, all strains differed from each other on FW, whereas on PM S1 exhibited superior growth compared to all other strains. On day 12, all pairwise contrasts between strains were significant except for the one between S3 and S4 on FW, and those between S2 and S3 and S4, respectively, on PM.

	Day	Diet	Strain-specific Contrast	Estimate	*P*
**a)**	0	PF	S1 – S2	-0.597	0.996
			S1 – S3	-0.115	1.000
			S1 – S4	-0.146	1.000
			S2 – S3	0.482	0.998
			S2 – S4	0.452	0.998
			S3 – S4	-0.030	1.000
	0	FW	S1 – S2	-0.521	0.997
			S1 – S3	-0.054	1.000
			S1 – S4	-0.094	1.000
			S2 – S3	0.467	0.998
			S2 – S4	0.428	0.998
			S3 – S4	-0.039	1.000
	0	PM	S1 – S2	-0.694	0.993
			S1 – S3	-0.123	1.000
			S1 – S4	-0.146	1.000
			S2 – S3	0.571	0.996
			S2 – S4	0.548	0.997
			S3 – S4	-0.023	1.000
**b)**	4	PF	S1 – S2	12.667	<.0001
			S1 – S3	11.833	<.0001
			S1 – S4	6.222	0.077
			S2 – S3	-0.834	0.988
			S2 – S4	-6.444	0.062
			S3 – S4	-5.611	0.133
	4	FW	S1 – S2	-7.778	0.015
			S1 – S3	-7.444	0.022
			S1 – S4	-15.556	<.0001
			S2 – S3	0.333	0.999
			S2 – S4	-7.778	0.015
			S3 – S4	-8.111	0.010
	4	PM	S1 – S2	4.722	0.261
			S1 – S3	2.167	0.835
			S1 – S4	-2.333	0.803
			S2 – S3	-2.556	0.755
			S2 – S4	-7.056	0.033
			S3 – S4	-4.500	0.303
**c)**	8	PF	S1 – S2	41.000	<.0001
			S1 – S3	14.056	<.0001
			S1 – S4	15.945	<.0001
			S2 – S3	-26.945	<.0001
			S2 – S4	-25.056	<.0001
			S3 – S4	1.889	0.884
	8	FW	S1 – S2	7.056	0.033
			S1 – S3	-27.500	<.0001
			S1 – S4	-5.222	0.182
			S2 – S3	-34.556	<.0001
			S2 – S4	-12.278	<.0001
			S3 – S4	22.278	<.0001
	8	PM	S1 – S2	14.389	<.0001
			S1 – S3	9.667	0.001
			S1 – S4	8.833	0.004
			S2 – S3	-4.722	0.261
			S2 – S4	-5.556	0.139
			S3 – S4	-0.834	0.988
**d)**	12	PF	S1 – S2	75.333	<.0001
			S1 – S3	48.222	<.0001
			S1 – S4	54.889	<.0001
			S2 – S3	-27.111	<.0001
			S2 – S4	-20.445	<.0001
			S3 – S4	6.666	0.050
	12	FW	S1 – S2	53.722	<.0001
			S1 – S3	12.667	<.0001
			S1 – S4	14.889	<.0001
			S2 – S3	-41.056	<.0001
			S2 – S4	-38.833	<.0001
			S3 – S4	2.223	0.825
	12	PM	S1 – S2	20.000	<.0001
			S1 – S3	14.222	<.0001
			S1 – S4	21.667	<.0001
			S2 – S3	-5.778	0.115
			S2 – S4	1.667	0.917
			S3 – S4	7.445	0.022

Table S7. Twofold interaction contrasts of pairwise strain and diet level comparisons for larval performance and body composition responses.

BSFL: black soldier fly larvae; DM: dry matter; N: nitrogen. Pairwise level combinations are indicated for the two factors strain (S1-4) and diet (poultry feed (PF), food waste (FW), poultry manure (PM)). Interaction contrast are based on one degree of freedom (DF) on 60 residual DFs throughout and values denote contrasts between pairwise contrasts for both factors (analogous to estimates). *P*-values were adjusted for multiple simultaneous testing.

Trait	Strain	Diet	Value	*F*	*P*	
a) BSFL live weight (mg) at harvest	S1-S2	PF-FW	0.039	4.79	0.228	
	S1-S3	PF-FW	0.182	101.81	<0.001	***
	S1-S4	PF-FW	0.149	57.96	<0.001	***
	S2-S3	PF-FW	0.143	77.43	<0.001	***
	S2-S4	PF-FW	0.111	37.77	<0.001	***
	S3-S4	PF-FW	-0.032	3.09	0.272	
	S1-S2	PF-PM	0.037	3.46	0.272	
	S1-S3	PF-PM	0.044	6.50	0.107	
	S1-S4	PF-PM	0.080	17.18	0.001	**
	S2-S3	PF-PM	0.007	0.16	1	
	S2-S4	PF-PM	0.043	4.77	0.228	
	S3-S4	PF-PM	0.036	4.40	0.228	
	S1-S2	FW-PM	-0.002	0.01	1	
	S1-S3	FW-PM	-0.138	80.84	<0.001	***
	S1-S4	FW-PM	-0.070	26.27	<0.001	***
	S2-S3	FW-PM	-0.136	43.28	<0.001	***
	S2-S4	FW-PM	-0.068	12.17	0.008	**
	S3-S4	FW-PM	0.069	22.96	<0.001	***
b) BSFL mortality (%)	S1-S2	PF-FW	-0.080	1.64	1	
	S1-S3	PF-FW	-0.039	0.45	1	
	S1-S4	PF-FW	-0.008	0.02	1	
	S2-S3	PF-FW	0.042	0.60	1	
	S2-S4	PF-FW	0.073	1.90	1	
	S3-S4	PF-FW	0.031	0.44	1	
	S1-S2	PF-PM	0.096	2.12	1	
	S1-S3	PF-PM	0.150	6.03	0.254	
	S1-S4	PF-PM	0.122	4.67	0.487	
	S2-S3	PF-PM	0.055	0.94	1	
	S2-S4	PF-PM	0.027	0.27	1	
	S3-S4	PF-PM	-0.028	0.37	1	
	S1-S2	FW-PM	0.176	17.09	0.002	**
	S1-S3	FW-PM	0.189	30.51	<0.001	***
	S1-S4	FW-PM	0.130	12.36	0.013	*
	S2-S3	FW-PM	0.013	0.09	1	
	S2-S4	FW-PM	-0.046	0.95	1	
	S3-S4	FW-PM	-0.059	2.21	1	
c) BSFL biomass (g DM)	S1-S2	PF-FW	-0.013	0.10	1	
	S1-S3	PF-FW	0.222	36.21	<0.001	***
	S1-S4	PF-FW	0.167	20.60	<0.001	***
	S2-S3	PF-FW	0.235	118.06	<0.001	***
	S2-S4	PF-FW	0.180	69.64	<0.001	***
	S3-S4	PF-FW	-0.055	12.57	0.009	**
	S1-S2	PF-PM	0.053	1.55	0.655	
	S1-S3	PF-PM	0.122	11.06	0.015	*
	S1-S4	PF-PM	0.114	10.02	0.019	*
	S2-S3	PF-PM	0.070	6.32	0.102	
	S2-S4	PF-PM	0.062	5.29	0.150	
	S3-S4	PF-PM	-0.008	0.24	1	
	S1-S2	FW-PM	0.065	4.16	0.229	
	S1-S3	FW-PM	-0.100	11.85	0.012	*
	S1-S4	FW-PM	-0.053	3.52	0.262	
	S2-S3	FW-PM	-0.165	62.12	<0.001	***
	S2-S4	FW-PM	-0.119	35.16	<0.001	***
	S3-S4	FW-PM	0.047	10.59	0.017	*
d) Estimated protein (g N×4.76/kg BSFL DM)	S1-S2	PF-FW	0.512	3.86	0.432	
	S1-S3	PF-FW	0.512	3.64	0.432	
	S1-S4	PF-FW	0.524	16.14	0.003	**
	S2-S3	PF-FW	0.500	0.00	1	
	S2-S4	PF-FW	0.512	4.30	0.387	
	S3-S4	PF-FW	0.513	4.47	0.387	
	S1-S2	PF-PM	0.487	5.01	0.319	
	S1-S3	PF-PM	0.510	3.05	0.516	
	S1-S4	PF-PM	0.501	0.06	1	
	S2-S3	PF-PM	0.523	16.05	0.003	**
	S2-S4	PF-PM	0.515	6.25	0.182	
	S3-S4	PF-PM	0.491	2.28	0.682	
	S1-S2	FW-PM	0.475	17.60	0.002	**
	S1-S3	FW-PM	0.499	0.05	1	
	S1-S4	FW-PM	0.477	14.66	0.004	**
	S2-S3	FW-PM	0.524	16.10	0.003	**
	S2-S4	FW-PM	0.502	0.15	1	
	S3-S4	FW-PM	0.479	13.27	0.007	**
e) Total estimated protein (g)	S1-S2	PF-FW	0.023	0.35	1	
	S1-S3	PF-FW	0.256	44.08	<0.001	***
	S1-S4	PF-FW	0.237	38.15	<0.001	***
	S2-S3	PF-FW	0.233	158.04	<0.001	***
	S2-S4	PF-FW	0.214	139.05	<0.001	***
	S3-S4	PF-FW	-0.019	1.60	1	
	S1-S2	PF-PM	0.015	0.13	1	
	S1-S3	PF-PM	0.147	16.28	0.001	**
	S1-S4	PF-PM	0.115	10.08	0.017	*
	S2-S3	PF-PM	0.132	28.56	<0.001	***
	S2-S4	PF-PM	0.101	16.83	0.001	**
	S3-S4	PF-PM	-0.032	4.52	0.226	
	S1-S2	FW-PM	-0.009	0.08	1	
	S1-S3	FW-PM	-0.109	15.96	0.001	**
	S1-S4	FW-PM	-0.122	19.28	0.001	***
	S2-S3	FW-PM	-0.100	27.16	<0.001	***
	S2-S4	FW-PM	-0.113	32.31	<0.001	***
	S3-S4	FW-PM	-0.013	1.55	1	
f) Estimated protein : ether extract ratio	S1-S2	PF-FW	0.002	0.00	1	
	S1-S3	PF-FW	0.478	11.99	0.008	**
	S1-S4	PF-FW	0.485	12.70	0.008	**
	S2-S3	PF-FW	0.477	32.91	<0.001	***
	S2-S4	PF-FW	0.484	36.77	<0.001	***
	S3-S4	PF-FW	0.007	0.04	1	
	S1-S2	PF-PM	-0.717	8.12	0.036	*
	S1-S3	PF-PM	0.220	2.30	0.537	
	S1-S4	PF-PM	0.483	10.04	0.017	*
	S2-S3	PF-PM	0.938	20.01	0.001	***
	S2-S4	PF-PM	1.201	31.26	<0.001	***
	S3-S4	PF-PM	0.263	17.84	0.001	**
	S1-S2	FW-PM	-0.719	12.47	0.008	**
	S1-S3	FW-PM	-0.258	18.36	0.001	***
	S1-S4	FW-PM	-0.002	0.00	1	
	S2-S3	FW-PM	0.461	5.23	0.129	
	S2-S4	FW-PM	0.717	12.19	0.008	**
	S3-S4	FW-PM	0.256	15.07	0.003	**

Table S7 continued.

Trait	Strain	Diet	Value	*F*	*P*	
g) Ether extract (g/kg BSFL DM)	S1-S2	PF-FW	0.503	0.01	1	
	S1-S3	PF-FW	0.344	23.14	<0.001	***
	S1-S4	PF-FW	0.358	19.04	<0.001	***
	S2-S3	PF-FW	0.341	23.06	<0.001	***
	S2-S4	PF-FW	0.355	19.04	<0.001	***
	S3-S4	PF-FW	0.515	0.22	1	
	S1-S2	PF-PM	0.711	23.70	<0.001	***
	S1-S3	PF-PM	0.425	2.46	0.490	
	S1-S4	PF-PM	0.348	9.89	0.023	*
	S2-S3	PF-PM	0.231	45.62	<0.001	***
	S2-S4	PF-PM	0.178	67.24	<0.001	***
	S3-S4	PF-PM	0.419	2.88	0.475	
	S1-S2	FW-PM	0.709	25.01	<0.001	***
	S1-S3	FW-PM	0.585	3.34	0.436	
	S1-S4	FW-PM	0.489	0.05	1	
	S2-S3	FW-PM	0.367	9.94	0.023	*
	S2-S4	FW-PM	0.282	26.43	<0.001	***
	S3-S4	FW-PM	0.404	4.10	0.332	
h) Total ether extract (g)	S1-S2	PF-FW	0.020	0.02	1	
	S1-S3	PF-FW	-0.226	3.10	0.417	
	S1-S4	PF-FW	-0.250	3.93	0.312	
	S2-S3	PF-FW	-0.245	8.26	0.052	.
	S2-S4	PF-FW	-0.269	10.78	0.021	*
	S3-S4	PF-FW	-0.024	0.40	1	
	S1-S2	PF-PM	0.731	9.38	0.036	*
	S1-S3	PF-PM	-0.075	0.30	1	
	S1-S4	PF-PM	-0.369	6.74	0.095	.
	S2-S3	PF-PM	-0.805	16.17	0.002	**
	S2-S4	PF-PM	-1.099	28.90	<0.001	***
	S3-S4	PF-PM	-0.294	25.47	<0.001	***
	S1-S2	FW-PM	0.711	13.02	0.008	**
	S1-S3	FW-PM	0.151	4.56	0.258	
	S1-S4	FW-PM	-0.119	2.46	0.488	
	S2-S3	FW-PM	-0.560	8.43	0.052	.
	S2-S4	FW-PM	-0.830	18.12	0.001	**
	S3-S4	FW-PM	-0.270	17.31	0.002	**
i) Total ash (g/kg BSFL DM)	S1-S2	PF-FW	0.572	49.78	<0.001	***
	S1-S3	PF-FW	0.583	68.50	<0.001	***
	S1-S4	PF-FW	0.601	102.83	<0.001	***
	S2-S3	PF-FW	0.512	1.41	0.721	
	S2-S4	PF-FW	0.530	9.57	0.021	*
	S3-S4	PF-FW	0.519	3.71	0.353	
	S1-S2	PF-PM	0.510	2.91	0.465	
	S1-S3	PF-PM	0.532	29.19	<0.001	***
	S1-S4	PF-PM	0.554	84.99	<0.001	***
	S2-S3	PF-PM	0.522	13.09	0.005	**
	S2-S4	PF-PM	0.544	54.40	<0.001	***
	S3-S4	PF-PM	0.523	14.42	0.003	**
	S1-S2	FW-PM	0.438	40.45	<0.001	***
	S1-S3	FW-PM	0.448	29.26	<0.001	***
	S1-S4	FW-PM	0.452	25.30	<0.001	***
	S2-S3	FW-PM	0.510	1.14	0.721	
	S2-S4	FW-PM	0.514	2.23	0.563	
	S3-S4	FW-PM	0.504	0.18	0.721	

Trait	Strain	Diet	Value	*F*	*P*	
j) Total ash (g)	S1-S2	PF-FW	0.259	29.23	<0.001	***
	S1-S3	PF-FW	0.539	175.63	<0.001	***
	S1-S4	PF-FW	0.552	205.07	<0.001	***
	S2-S3	PF-FW	0.280	49.56	<0.001	***
	S2-S4	PF-FW	0.294	60.85	<0.001	***
	S3-S4	PF-FW	0.014	0.24	0.625	
	S1-S2	PF-PM	0.096	4.86	0.063	.
	S1-S3	PF-PM	0.233	44.87	<0.001	***
	S1-S4	PF-PM	0.301	80.35	<0.001	***
	S2-S3	PF-PM	0.137	12.49	0.004	**
	S2-S4	PF-PM	0.205	29.46	<0.001	***
	S3-S4	PF-PM	0.068	6.06	0.050	.
	S1-S2	FW-PM	-0.163	24.66	<0.001	***
	S1-S3	FW-PM	-0.306	94.61	<0.001	***
	S1-S4	FW-PM	-0.252	63.17	<0.001	***
	S2-S3	FW-PM	-0.143	60.51	<0.001	***
	S2-S4	FW-PM	-0.089	22.38	<0.001	***
	S3-S4	FW-PM	0.054	10.96	0.006	**
k) Bioconversion efficiency (g BSFL DM/kg diet DM)	S1-S2	PF-FW	0.020	2.45	0.475	
	S1-S3	PF-FW	0.078	41.20	<0.001	***
	S1-S4	PF-FW	0.065	28.40	<0.001	***
	S2-S3	PF-FW	0.058	84.81	<0.001	***
	S2-S4	PF-FW	0.045	50.77	<0.001	***
	S3-S4	PF-FW	-0.013	9.96	0.017	*
	S1-S2	PF-PM	0.060	23.16	<0.001	***
	S1-S3	PF-PM	0.075	43.80	<0.001	***
	S1-S4	PF-PM	0.075	44.10	<0.001	***
	S2-S3	PF-PM	0.015	5.34	0.141	
	S2-S4	PF-PM	0.015	5.41	0.141	
	S3-S4	PF-PM	0.000	0.002	1	
	S1-S2	FW-PM	0.039	36.77	<0.001	***
	S1-S3	FW-PM	-0.003	0.31	1	
	S1-S4	FW-PM	0.010	2.51	0.475	
	S2-S3	FW-PM	-0.043	161.52	<0.001	***
	S2-S4	FW-PM	-0.030	80.65	<0.001	***
	S3-S4	FW-PM	0.013	23.17	<0.001	***
l) Nitrogen efficiency (g N BSFL/kg N diet)	S1-S2	PF-FW	0.046	3.11	0.496	
	S1-S3	PF-FW	0.159	39.47	<0.001	***
	S1-S4	PF-FW	0.149	35.07	<0.001	***
	S2-S3	PF-FW	0.113	133.07	<0.001	***
	S2-S4	PF-FW	0.103	116.40	<0.001	***
	S3-S4	PF-FW	-0.010	1.56	0.865	
	S1-S2	PF-PM	0.103	18.73	<0.001	***
	S1-S3	PF-PM	0.149	41.95	<0.001	***
	S1-S4	PF-PM	0.139	36.96	<0.001	***
	S2-S3	PF-PM	0.046	21.77	<0.001	***
	S2-S4	PF-PM	0.036	14.26	0.003	**
	S3-S4	PF-PM	-0.010	2.12	0.755	
	S1-S2	FW-PM	0.057	23.22	<0.001	***
	S1-S3	FW-PM	-0.010	0.75	1	
	S1-S4	FW-PM	-0.010	0.80	1	
	S2-S3	FW-PM	-0.067	214.28	<0.001	***
	S2-S4	FW-PM	-0.068	183.87	<0.001	***
	S3-S4	FW-PM	0.000	0.02	1	

Table S7 continued.

	Trait	Strain	Diet	Value	*F*	*P*	
m)	Dry matter reduction (g/kg diet)	S1-S2	PF-FW	0.029	1.43	0.692	
		S1-S3	PF-FW	0.025	4.21	0.268	
		S1-S4	PF-FW	0.076	29.15	<0.001	***
		S2-S3	PF-FW	-0.004	0.03	0.860	
		S2-S4	PF-FW	0.048	4.09	0.268	
		S3-S4	PF-FW	0.052	22.22	<0.001	***
		S1-S2	PF-PM	0.097	37.15	<0.001	***
		S1-S3	PF-PM	0.014	1.47	0.692	
		S1-S4	PF-PM	0.056	19.01	0.001	***
		S2-S3	PF-PM	-0.083	37.07	<0.001	***
		S2-S4	PF-PM	-0.042	8.35	0.048	*
		S3-S4	PF-PM	0.041	18.20	0.001	***
		S1-S2	FW-PM	0.069	12.74	0.007	**
		S1-S3	FW-PM	-0.010	4.49	0.268	
		S1-S4	FW-PM	-0.021	6.91	0.087	.
		S2-S3	FW-PM	-0.079	17.48	0.001	**
		S2-S4	FW-PM	-0.089	20.17	<0.001	***
		S3-S4	FW-PM	-0.010	2.08	0.616	
n)	Neutral detergent fibre reduction (g/kg diet)	S1-S2	PF-FW	-0.029	1.61	1	
		S1-S3	PF-FW	0.004	0.02	1	
		S1-S4	PF-FW	0.020	0.48	1	
		S2-S3	PF-FW	0.033	1.16	1	
		S2-S4	PF-FW	0.049	3.20	1	
		S3-S4	PF-FW	0.016	0.20	1	
		S1-S2	PF-PM	-0.022	0.40	1	
		S1-S3	PF-PM	0.049	1.32	1	
		S1-S4	PF-PM	-0.020	0.40	1	
		S2-S3	PF-PM	0.071	3.32	1	
		S2-S4	PF-PM	0.002	0.01	1	
		S3-S4	PF-PM	-0.069	3.57	1	
		S1-S2	FW-PM	0.007	0.04	1	
		S1-S3	FW-PM	0.045	2.06	1	
		S1-S4	FW-PM	-0.040	1.43	1	
		S2-S3	FW-PM	0.038	1.47	1	
		S2-S4	FW-PM	-0.047	1.93	1	
		S3-S4	FW-PM	-0.085	6.79	0.208	
o)	Acid detergent fibre reduction (g/kg diet)	S1-S2	PF-FW	0.017	0.16	1	
		S1-S3	PF-FW	0.002	0.001	1	
		S1-S4	PF-FW	0.076	2.47	1	
		S2-S3	PF-FW	-0.015	0.10	1	
		S2-S4	PF-FW	0.059	1.33	1	
		S3-S4	PF-FW	0.074	1.98	1	
		S1-S2	PF-PM	0.036	0.57	1	
		S1-S3	PF-PM	0.069	1.11	1	
		S1-S4	PF-PM	0.066	2.43	1	
		S2-S3	PF-PM	0.032	0.29	1	
		S2-S4	PF-PM	0.030	0.74	1	
		S3-S4	PF-PM	-0.003	0.002	1	
		S1-S2	FW-PM	0.020	0.15	1	
		S1-S3	FW-PM	0.067	1.48	1	
		S1-S4	FW-PM	-0.010	0.03	1	
		S2-S3	FW-PM	0.048	0.66	1	
		S2-S4	FW-PM	-0.029	0.28	1	
		S3-S4	FW-PM	-0.077	1.62	1	
p)	Hemicellulose reduction (g/kg diet)	S1-S2	PF-FW	-0.110	9.10	0.045	*
		S1-S3	PF-FW	-0.003	0.01	1	
		S1-S4	PF-FW	-0.058	2.58	0.769	
		S2-S3	PF-FW	0.107	10.52	0.025	*
		S2-S4	PF-FW	0.052	3.80	0.504	
		S3-S4	PF-FW	-0.055	2.86	0.769	
		S1-S2	PF-PM	-0.116	7.19	0.094	.
		S1-S3	PF-PM	-0.029	0.25	1	
		S1-S4	PF-PM	-0.228	33.75	<0.001	***
		S2-S3	PF-PM	0.087	2.73	0.769	
		S2-S4	PF-PM	-0.112	13.58	0.007	**
		S3-S4	PF-PM	-0.199	16.11	0.003	**
		S1-S2	FW-PM	-0.007	0.02	1	
		S1-S3	FW-PM	-0.026	0.18	1	
		S1-S4	FW-PM	-0.170	12.10	0.013	*
		S2-S3	FW-PM	-0.019	0.16	1	
		S2-S4	FW-PM	-0.164	29.70	<0.001	***
		S3-S4	FW-PM	-0.144	8.94	0.045	*
q)	Dry matter losses (g emissions / kg diet)	S1-S2	PF-FW	0.530	5.97	0.140	
		S1-S3	PF-FW	0.438	27.29	<0.001	***
		S1-S4	PF-FW	0.498	0.02	1	
		S2-S3	PF-FW	0.409	58.71	<0.001	***
		S2-S4	PF-FW	0.469	6.71	0.120	
		S3-S4	PF-FW	0.560	26.34	<0.001	***
		S1-S2	PF-PM	0.534	7.31	0.098	
		S1-S3	PF-PM	0.429	32.57	<0.001	***
		S1-S4	PF-PM	0.473	4.79	0.228	.
		S2-S3	PF-PM	0.396	71.14	<0.001	***
		S2-S4	PF-PM	0.439	24.33	<0.001	***
		S3-S4	PF-PM	0.544	12.89	0.008	**
		S1-S2	FW-PM	0.505	0.16	1	
		S1-S3	FW-PM	0.491	0.55	1	
		S1-S4	FW-PM	0.474	4.48	0.231	
		S2-S3	FW-PM	0.486	1.31	1	
		S2-S4	FW-PM	0.469	6.36	0.129	
		S3-S4	FW-PM	0.483	1.91	0.858	

Table S8. Pairwise contrasts between strains within dietary levels for larval performance and body composition responses.

BSFL: black soldier fly larvae; DM: dry matter; N: nitrogen. Pairwise strain-specific contrasts (S1-4) were performed within fixed dietary levels (poultry feed (PF), food waste (FW), poultry manure (PM)). Estimates are reported on specifically transformed or canonical link scales, respectively (see Table 3 for details). *P*-values were adjusted for multiple simultaneous testing.

	Trait	Diet	Strain-specific Contrast	Estimate	*P*		Trait	Diet	Strain-specific Contrast	Estimate	*P*
a)	BSFL live weight (mg) at harvest	PF	S1 – S2	0.332	<0.001	d)	Estimated protein (g N×4.76/kg BSFL DM)	PF	S1 – S2	-0.089	<0.001
			S1 – S3	0.226	<0.001				S1 – S3	-0.045	0.041
			S1 – S4	0.237	<0.001				S1 – S4	-0.073	0.000
			S2 – S3	-0.108	<0.001				S2 – S3	0.045	0.038
			S2 – S4	-0.096	<0.001				S2 – S4	0.016	0.760
			S3 – S4	0.010	0.899				S3 – S4	-0.028	0.331
		FW	S1 – S2	0.294	<0.001			FW	S1 – S2	-0.137	<0.001
			S1 – S3	0.045	0.002				S1 – S3	-0.091	<0.001
			S1 – S4	0.087	<0.001				S1 – S4	-0.169	<0.001
			S2 – S3	-0.249	<0.001				S2 – S3	0.046	0.037
			S2 – S4	-0.206	<0.001				S2 – S4	-0.033	0.210
			S3 – S4	0.043	0.002				S3 – S4	-0.079	<.0001
		PM	S1 – S2	0.295	<0.001			PM	S1 – S2	-0.037	0.113
			S1 – S3	0.183	<0.001				S1 – S3	-0.086	<0.001
			S1 – S4	0.157	<0.001				S1 – S4	-0.079	<0.001
			S2 – S3	-0.113	<0.001				S2 – S3	-0.049	0.014
			S2 – S4	-0.139	<0.001				S2 – S4	-0.042	0.050
			S3 – S4	-0.026	0.026				S3 – S4	0.007	0.974

	Trait	Diet	Strain-specific Contrast	Estimate	*P*		Trait	Diet	Strain-specific Contrast	Estimate	*P*
b)	BSFL mortality (%)	PF	S1 – S2	0.086	0.439	e)	Total estimated protein (g)	PF	S1 – S2	0.224	<0.001
			S1 – S3	0.160	0.024				S1 – S3	0.287	<0.001
			S1 – S4	0.142	0.032				S1 – S4	0.274	<0.001
			S2 – S3	0.074	0.364				S2 – S3	0.063	0.015
			S2 – S4	0.057	0.501				S2 – S4	0.050	0.076
			S3 – S4	-0.018	0.963				S3 – S4	-0.013	0.915
		FW	S1 – S2	0.166	<0.001			FW	S1 – S2	0.201	<0.001
			S1 – S3	0.199	<0.001				S1 – S3	0.031	0.428
			S1 – S4	0.150	<0.001				S1 – S4	0.037	0.277
			S2 – S3	0.033	0.688				S2 – S3	-0.170	<0.001
			S2 – S4	-0.016	0.964				S2 – S4	-0.164	<0.001
			S3 – S4	-0.049	0.333				S3 – S4	0.006	0.992
		PM	S1 – S2	-0.010	0.990			PM	S1 – S2	0.210	<0.001
			S1 – S3	0.010	0.986				S1 – S3	0.140	<0.001
			S1 – S4	0.020	0.873				S1 – S4	0.159	<0.001
			S2 – S3	0.019	0.940				S2 – S3	-0.070	0.006
			S2 – S4	0.030	0.797				S2 – S4	-0.051	0.068
			S3 – S4	0.010	0.982				S3 – S4	0.019	0.791

	Trait	Diet	Strain-specific Contrast	Estimate	*P*		Trait	Diet	Strain-specific Contrast	Estimate	*P*
c)	BSFL biomass (g DM)	PF	S1 – S2	0.286	<0.001	f)	Estimated protein : ether extract ratio	PF	S1 – S2	-0.222	0.465
			S1 – S3	0.318	<0.001				S1 – S3	0.317	0.100
			S1 – S4	0.324	<0.001				S1 – S4	0.336	0.074
			S2 – S3	0.032	0.373				S2 – S3	0.539	<0.001
			S2 – S4	0.039	0.216				S2 – S4	0.558	<0.001
			S3 – S4	0.006	0.955				S3 – S4	0.019	0.814
		FW	S1 – S2	0.298	<0.001			FW	S1 – S2	-0.224	<0.001
			S1 – S3	0.096	<0.001				S1 – S3	-0.162	<0.001
			S1 – S4	0.157	<0.001				S1 – S4	-0.150	<0.001
			S2 – S3	-0.202	<0.001				S2 – S3	0.062	0.462
			S2 – S4	-0.141	<0.001				S2 – S4	0.074	0.156
			S3 – S4	0.061	<0.001				S3 – S4	0.012	0.981
		PM	S1 – S2	0.233	<0.001			PM	S1 – S2	0.495	0.076
			S1 – S3	0.196	<0.001				S1 – S3	0.097	0.275
			S1 – S4	0.210	<0.001				S1 – S4	-0.148	0.164
			S2 – S3	-0.037	0.224				S2 – S3	-0.398	0.191
			S2 – S4	-0.023	0.587				S2 – S4	-0.643	0.012
			S3 – S4	0.014	0.549				S3 – S4	-0.245	0.001

Table S8 continued.

	Trait	Diet	Strain-specific Contrast	Estimate	*P*
**g)**	Ether extract (g/kg BSFL DM)	PF	S1 – S2	0.249	0.084
			S1 – S3	-0.463	<0.001
			S1 – S4	-0.524	<0.001
			S2 – S3	-0.712	<0.001
			S2 – S4	-0.772	<0.001
			S3 – S4	-0.060	0.918
		FW	S1 – S2	0.237	0.049
			S1 – S3	0.183	0.190
			S1 – S4	0.061	0.910
			S2 – S3	-0.054	0.936
			S2 – S4	-0.176	0.222
			S3 – S4	-0.122	0.544
		PM	S1 – S2	-0.653	<0.001
			S1 – S3	-0.162	0.760
			S1 – S4	0.106	0.930
			S2 – S3	0.491	0.004
			S2 – S4	0.759	<0.001
			S3 – S4	0.268	0.389
**h)**	Total ether extract (g)	PF	S1 – S2	0.446	0.013
			S1 – S3	-0.032	0.994
			S1 – S4	-0.062	0.958
			S2 – S3	-0.477	<0.001
			S2 – S4	-0.508	<0.001
			S3 – S4	-0.030	0.336
		FW	S1 – S2	0.426	<0.001
			S1 – S3	0.194	<0.001
			S1 – S4	0.188	<0.001
			S2 – S3	-0.232	<0.001
			S2 – S4	-0.238	<0.001
			S3 – S4	-0.006	0.998
		PM	S1 – S2	-0.285	0.455
			S1 – S3	0.043	0.885
			S1 – S4	0.307	<0.001
			S2 – S3	0.328	0.306
			S2 – S4	0.592	0.015
			S3 – S4	0.264	<0.001
**i)**	Total ash (g/kg BSFL DM)	PF	S1 – S2	0.115	<0.001
			S1 – S3	0.106	<0.001
			S1 – S4	0.157	<0.001
			S2 – S3	-0.009	0.968
			S2 – S4	0.042	0.128
			S3 – S4	0.051	0.041
		FW	S1 – S2	-0.173	<0.001
			S1 – S3	-0.228	<0.001
			S1 – S4	-0.252	<0.001
			S2 – S3	-0.055	0.371
			S2 – S4	-0.079	0.094
			S3 – S4	-0.024	0.894
		PM	S1 – S2	0.075	<0.001
			S1 – S3	-0.020	0.483
			S1 – S4	-0.060	<0.001
			S2 – S3	-0.095	<0.001
			S2 – S4	-0.134	<0.001
			S3 – S4	-0.039	0.024

	Trait	Diet	Strain-specific Contrast	Estimate	*P*
**j)**	Total ash (g)	PF	S1 – S2	0.388	<0.001
			S1 – S3	0.413	<0.001
			S1 – S4	0.464	<0.001
			S2 – S3	0.025	0.912
			S2 – S4	0.076	0.148
			S3 – S4	0.052	0.184
		FW	S1 – S2	0.130	<0.001
			S1 – S3	-0.126	<0.001
			S1 – S4	-0.088	0.007
			S2 – S3	-0.256	<0.001
			S2 – S4	-0.218	<0.001
			S3 – S4	0.038	0.014
		PM	S1 – S2	0.293	<0.001
			S1 – S3	0.180	<0.001
			S1 – S4	0.164	<0.001
			S2 – S3	-0.113	<0.001
			S2 – S4	-0.129	<0.001
			S3 – S4	-0.016	0.457
**k)**	Bioconversion efficiency (g BSFL DM/kg diet DM)	PF	S1 – S2	0.095	<0.001
			S1 – S3	0.104	<0.001
			S1 – S4	0.106	<0.001
			S2 – S3	0.009	0.442
			S2 – S4	0.011	0.265
			S3 – S4	0.002	0.951
		FW	S1 – S2	0.074	<0.001
			S1 – S3	0.025	<0.001
			S1 – S4	0.041	<0.001
			S2 – S3	-0.049	<0.001
			S2 – S4	-0.034	<0.001
			S3 – S4	0.015	<0.001
		PM	S1 – S2	0.035	<0.001
			S1 – S3	0.029	<0.001
			S1 – S4	0.031	<0.001
			S2 – S3	-0.006	0.112
			S2 – S4	-0.004	0.384
			S3 – S4	0.002	0.509
**l)**	Nitrogen efficiency (g N BSFL/kg N diet)	PF	S1 – S2	0.144	<0.001
			S1 – S3	0.176	<0.001
			S1 – S4	0.169	<0.001
			S2 – S3	0.032	0.006
			S2 – S4	0.025	0.030
			S3 – S4	-0.007	0.766
		FW	S1 – S2	0.098	<0.001
			S1 – S3	0.017	0.434
			S1 – S4	0.020	0.300
			S2 – S3	-0.081	<0.001
			S2 – S4	-0.078	<0.001
			S3 – S4	0.003	0.836
		PM	S1 – S2	0.041	<0.001
			S1 – S3	0.027	<0.001
			S1 – S4	0.030	<0.001
			S2 – S3	-0.014	<0.001
			S2 – S4	-0.010	0.013
			S3 – S4	0.004	0.059

Table S8 continued.

	Trait	Diet	Strain-specific Contrast	Estimate	***P***
**m)**	Dry matter reduction (g/kg diet)	PF	S1 – S2	0.113	<0.001
			S1 – S3	0.007	0.919
			S1 – S4	0.039	0.013
			S2 – S3	-0.106	<0.001
			S2 – S4	-0.074	<0.001
			S3 – S4	0.031	0.005
		FW	S1 – S2	0.084	<0.001
			S1 – S3	-0.018	<0.001
			S1 – S4	-0.038	<0.001
			S2 – S3	-0.102	<0.001
			S2 – S4	-0.122	<0.001
			S3 – S4	-0.020	0.009
		PM	S1 – S2	0.015	0.009
			S1 – S3	-0.007	0.114
			S1 – S4	-0.017	<0.001
			S2 – S3	-0.023	<0.001
			S2 – S4	-0.033	<0.001
			S3 – S4	-0.010	0.027

	Trait	Diet	Strain-specific Contrast	Estimate	***P***
**n)**	Neutral detergent fibre reduction (g/kg diet)	PF	S1 – S2	-0.004	0.995
			S1 – S3	-0.006	0.998
			S1 – S4	0.009	0.967
			S2 – S3	-0.001	0.999
			S2 – S4	0.013	0.712
			S3 – S4	0.014	0.953
		FW	S1 – S2	0.024	0.291
			S1 – S3	-0.010	0.718
			S1 – S4	-0.011	0.955
			S2 – S3	-0.034	0.087
			S2 – S4	-0.036	0.464
			S3 – S4	-0.001	0.999
		PM	S1 – S2	0.018	0.932
			S1 – S3	-0.055	0.266
			S1 – S4	0.029	0.671
			S2 – S3	-0.072	0.058
			S2 – S4	0.011	0.963
			S3 – S4	0.083	0.005

	Trait	Diet	Strain-specific Contrast	Estimate	***P***
**o)**	Acid detergent fibre reduction (g/kg diet)	PF	S1 – S2	0.023	0.846
			S1 – S3	-0.020	0.961
			S1 – S4	0.018	0.907
			S2 – S3	-0.043	0.613
			S2 – S4	-0.005	0.992
			S3 – S4	0.038	0.678
		FW	S1 – S2	0.006	0.997
			S1 – S3	-0.021	0.675
			S1 – S4	-0.058	0.481
			S2 – S3	-0.028	0.816
			S2 – S4	-0.064	0.534
			S3 – S4	-0.036	0.805
		PM	S1 – S2	-0.013	0.987
			S1 – S3	-0.088	0.330
			S1 – S4	-0.048	0.466
			S2 – S3	-0.075	0.429
			S2 – S4	-0.035	0.620
			S3 – S4	0.040	0.804

	Trait	Diet	Strain-specific Contrast	Estimate	***P***
**p)**	Hemicellulose reduction (g/kg diet)	PF	S1 – S2	-0.029	0.448
			S1 – S3	0.006	0.995
			S1 – S4	0.001	1
			S2 – S3	0.035	0.586
			S2 – S4	0.030	0.402
			S3 – S4	-0.005	0.997
		FW	S1 – S2	0.081	0.052
			S1 – S3	0.009	0.993
			S1 – S4	0.059	0.290
			S2 – S3	-0.072	0.001
			S2 – S4	-0.022	0.641
			S3 – S4	0.050	0.103
		PM	S1 – S2	0.087	0.121
			S1 – S3	0.035	0.913
			S1 – S4	0.229	<0.001
			S2 – S3	-0.053	0.653
			S2 – S4	0.142	<0.001
			S3 – S4	0.194	<0.001

	Trait	Diet	Strain-specific Contrast	Estimate	***P***
**q)**	Dry matter losses (g emissions / kg diet)	PF	S1 – S2	0.121	0.004
			S1 – S3	-0.392	<0.001
			S1 – S4	-0.268	<0.001
			S2 – S3	-0.513	<0.001
			S2 – S4	-0.389	<0.001
			S3 – S4	0.124	0.001
		FW	S1 – S2	0.002	0.999
			S1 – S3	-0.144	<0.001
			S1 – S4	-0.262	<0.001
			S2 – S3	-0.146	<0.001
			S2 – S4	-0.264	<0.001
			S3 – S4	-0.118	0.002
		PM	S1 – S2	-0.017	0.965
			S1 – S3	-0.108	0.014
			S1 – S4	-0.159	<0.001
			S2 – S3	-0.091	0.054
			S2 – S4	-0.142	<0.001
			S3 – S4	-0.051	0.474

Table S9. Twofold interaction contrasts of pairwise strain and diet level comparisons for larval amino acid profiles (g/100 g protein).

AA: amino acid (according to conventional abbreviations). Pairwise level combinations are indicated for the two factors strain (S1-4) and diet (poultry feed (PF), food waste (FW), poultry manure (PM)). Interaction contrast are based on one degree of freedom (DF) on 60 residual DFs throughout and values denote contrasts between pairwise contrasts for both factors (analogous to estimates). *P*-values were adjusted for multiple simultaneous testing.

AA	Strain	Diet	Value	*F*	*P*	
a) *His*	S1-S2	PF-FW	-0.002	0.00	1	
	S1-S3	PF-FW	-0.109	4.68	0.415	
	S1-S4	PF-FW	-0.087	2.22	1	
	S2-S3	PF-FW	-0.107	8.41	0.078	.
	S2-S4	PF-FW	-0.086	3.24	0.844	
	S3-S4	PF-FW	0.021	0.17	1	
	S1-S2	PF-PM	0.145	4.85	0.410	
	S1-S3	PF-PM	-0.039	0.35	1	
	S1-S4	PF-PM	-0.085	1.47	1	
	S2-S3	PF-PM	-0.185	13.06	0.011	*
	S2-S4	PF-PM	-0.231	17.27	0.002	**
	S3-S4	PF-PM	-0.046	0.66	1	
	S1-S2	FW-PM	0.147	5.49	0.314	
	S1-S3	FW-PM	0.069	1.29	1	
	S1-S4	FW-PM	0.002	0.00	1	
	S2-S3	FW-PM	-0.078	2.56	1	
	S2-S4	FW-PM	-0.145	9.21	0.057	.
	S3-S4	FW-PM	-0.067	2.16	1	
b) *Ile*	S1-S2	PF-FW	0.014	0.02	1	
	S1-S3	PF-FW	0.066	0.36	1	
	S1-S4	PF-FW	-0.077	0.23	1	
	S2-S3	PF-FW	0.051	0.27	1	
	S2-S4	PF-FW	-0.091	0.35	1	
	S3-S4	PF-FW	-0.143	0.83	1	
	S1-S2	PF-PM	0.027	0.06	1	
	S1-S3	PF-PM	0.017	0.01	1	
	S1-S4	PF-PM	0.140	0.64	1	
	S2-S3	PF-PM	-0.010	0.00	1	
	S2-S4	PF-PM	0.113	0.58	1	
	S3-S4	PF-PM	0.123	0.34	1	
	S1-S2	FW-PM	0.012	0.01	1	
	S1-S3	FW-PM	-0.049	0.08	1	
	S1-S4	FW-PM	0.217	2.70	1	
	S2-S3	FW-PM	-0.062	0.13	1	
	S2-S4	FW-PM	0.204	2.69	1	
	S3-S4	FW-PM	0.266	2.06	1	
c) *Leu*	S1-S2	PF-FW	-0.129	1.46	1	
	S1-S3	PF-FW	-0.172	3.20	1	
	S1-S4	PF-FW	-0.359	11.42	0.023	*
	S2-S3	PF-FW	-0.044	0.31	1	
	S2-S4	PF-FW	-0.231	6.41	0.210	
	S3-S4	PF-FW	-0.187	5.54	0.307	
	S1-S2	PF-PM	0.056	0.61	1	
	S1-S3	PF-PM	-0.019	0.05	1	
	S1-S4	PF-PM	-0.197	6.58	0.206	
	S2-S3	PF-PM	-0.075	0.58	1	
	S2-S4	PF-PM	-0.253	8.15	0.100	
	S3-S4	PF-PM	-0.178	3.05	1	
	S1-S2	FW-PM	0.184	2.75	1	
	S1-S3	FW-PM	0.154	1.69	1	
	S1-S4	FW-PM	0.163	2.52	1	
	S2-S3	FW-PM	-0.031	0.10	1	
	S2-S4	FW-PM	-0.022	0.08	1	
	S3-S4	FW-PM	0.009	0.01	1	

AA	Strain	Diet	Value	*F*	*P*	
d) *Lys*	S1-S2	PF-FW	-0.067	0.35	1	
	S1-S3	PF-FW	-0.607	28.54	<0.001	***
	S1-S4	PF-FW	-0.364	10.26	0.033	*
	S2-S3	PF-FW	-0.540	22.55	<0.001	***
	S2-S4	PF-FW	-0.297	6.81	0.133	
	S3-S4	PF-FW	0.243	4.58	0.328	
	S1-S2	PF-PM	-0.038	0.11	1	
	S1-S3	PF-PM	-0.368	10.51	0.031	*
	S1-S4	PF-PM	-0.066	0.34	1	
	S2-S3	PF-PM	-0.330	8.44	0.072	.
	S2-S4	PF-PM	-0.028	0.06	1	
	S3-S4	PF-PM	0.302	7.08	0.130	
	S1-S2	FW-PM	0.029	0.07	1	
	S1-S3	FW-PM	0.239	4.41	0.328	
	S1-S4	FW-PM	0.298	6.88	0.133	
	S2-S3	FW-PM	0.210	3.40	0.491	
	S2-S4	FW-PM	0.269	5.60	0.212	
	S3-S4	FW-PM	0.059	0.27	1	
e) *Met*	S1-S2	PF-FW	-0.275	46.00	<0.001	***
	S1-S3	PF-FW	-0.400	66.46	<0.001	***
	S1-S4	PF-FW	-0.270	17.85	0.001	***
	S2-S3	PF-FW	-0.125	8.84	0.042	*
	S2-S4	PF-FW	0.006	0.01	1	
	S3-S4	PF-FW	0.130	4.06	0.338	
	S1-S2	PF-PM	-0.056	1.14	0.868	
	S1-S3	PF-PM	0.106	3.89	0.338	
	S1-S4	PF-PM	0.120	3.26	0.380	
	S2-S3	PF-PM	0.161	13.24	0.006	**
	S2-S4	PF-PM	0.175	8.80	0.042	*
	S3-S4	PF-PM	0.014	0.05	1	
	S1-S2	FW-PM	0.220	20.37	<0.001	***
	S1-S3	FW-PM	0.506	69.64	<0.001	***
	S1-S4	FW-PM	0.389	33.10	<0.001	***
	S2-S3	FW-PM	0.286	31.57	<0.001	***
	S2-S4	FW-PM	0.170	8.23	0.045	*
	S3-S4	FW-PM	-0.116	2.82	0.393	
f) *Phe*	S1-S2	PF-FW	-0.214	8.03	0.074	.
	S1-S3	PF-FW	-0.138	3.57	0.510	
	S1-S4	PF-FW	-0.129	3.16	0.565	
	S2-S3	PF-FW	0.076	1.53	1	
	S2-S4	PF-FW	0.085	1.95	1	
	S3-S4	PF-FW	0.009	0.02	1	
	S1-S2	PF-PM	-0.150	7.07	0.101	
	S1-S3	PF-PM	0.162	4.52	0.339	
	S1-S4	PF-PM	0.200	8.07	0.074	.
	S2-S3	PF-PM	0.312	17.16	0.002	**
	S2-S4	PF-PM	0.350	25.29	<0.001	***
	S3-S4	PF-PM	0.038	0.20	1	
	S1-S2	FW-PM	0.064	0.63	1	
	S1-S3	FW-PM	0.299	11.24	0.019	*
	S1-S4	FW-PM	0.329	17.88	0.001	**
	S2-S3	FW-PM	0.236	8.55	0.063	.
	S2-S4	FW-PM	0.265	15.37	0.003	**
	S3-S4	FW-PM	0.029	0.14	1	

Table S9 continued.

AA	Strain	Diet	Value	*F*	*P*	
g) *Thr*	S1-S2	PF-FW	-0.101	1.67	1	
	S1-S3	PF-FW	-0.082	1.92	1	
	S1-S4	PF-FW	-0.108	1.75	1	
	S2-S3	PF-FW	0.019	0.11	1	
	S2-S4	PF-FW	-0.007	0.01	1	
	S3-S4	PF-FW	-0.026	0.17	1	
	S1-S2	PF-PM	-0.101	4.92	0.335	
	S1-S3	PF-PM	0.089	1.86	1	
	S1-S4	PF-PM	0.194	8.04	0.087	.
	S2-S3	PF-PM	0.190	10.98	0.023	*
	S2-S4	PF-PM	0.295	23.23	<0.001	***
	S3-S4	PF-PM	0.105	1.89	1	
	S1-S2	FW-PM	0.000	0.00	1	
	S1-S3	FW-PM	0.171	5.49	0.293	
	S1-S4	FW-PM	0.302	18.81	0.001	***
	S2-S3	FW-PM	0.171	5.48	0.293	
	S2-S4	FW-PM	0.302	18.76	0.001	***
	S3-S4	FW-PM	0.131	3.94	0.518	
h) *Val*	S1-S2	PF-FW	-0.568	12.78	0.012	*
	S1-S3	PF-FW	-0.541	15.67	0.004	**
	S1-S4	PF-FW	-0.268	4.93	0.303	
	S2-S3	PF-FW	0.026	0.03	1	
	S2-S4	PF-FW	0.299	4.72	0.304	
	S3-S4	PF-FW	0.273	5.98	0.206	
	S1-S2	PF-PM	0.007	0.00	1	
	S1-S3	PF-PM	-0.428	8.44	0.072	.
	S1-S4	PF-PM	-0.215	2.31	0.803	
	S2-S3	PF-PM	-0.435	10.56	0.030	*
	S2-S4	PF-PM	-0.222	3.03	0.609	
	S3-S4	PF-PM	0.213	3.98	0.406	
	S1-S2	FW-PM	0.574	8.92	0.061	.
	S1-S3	FW-PM	0.113	0.56	1	
	S1-S4	FW-PM	0.053	0.14	1	
	S2-S3	FW-PM	-0.461	6.01	0.206	
	S2-S4	FW-PM	-0.521	8.36	0.072	.
	S3-S4	FW-PM	-0.060	0.20	1	
i) *Ala*	S1-S2	PF-FW	-0.685	14.94	0.004	**
	S1-S3	PF-FW	-0.155	0.79	1	
	S1-S4	PF-FW	-0.455	7.21	0.075	.
	S2-S3	PF-FW	0.530	16.27	0.002	**
	S2-S4	PF-FW	0.230	3.31	0.369	
	S3-S4	PF-FW	-0.301	6.18	0.110	
	S1-S2	PF-PM	0.027	0.02	1	
	S1-S3	PF-PM	0.462	4.54	0.224	
	S1-S4	PF-PM	0.706	13.50	0.007	**
	S2-S3	PF-PM	0.435	7.99	0.057	.
	S2-S4	PF-PM	0.679	34.10	<0.001	***
	S3-S4	PF-PM	0.244	2.83	0.391	
	S1-S2	FW-PM	0.712	19.99	0.001	***
	S1-S3	FW-PM	0.617	11.26	0.017	*
	S1-S4	FW-PM	1.161	65.73	<0.001	***
	S2-S3	FW-PM	-0.095	0.26	1	
	S2-S4	FW-PM	0.449	9.63	0.029	*
	S3-S4	FW-PM	0.544	10.09	0.026	*

AA	Strain	Diet	Value	*F*	*P*	
j) *Asx (Asp + Asn)*	S1-S2	PF-FW	-0.390	6.48	0.162	
	S1-S3	PF-FW	0.385	6.31	0.162	
	S1-S4	PF-FW	0.041	0.07	1	
	S2-S3	PF-FW	0.774	25.59	<0.001	***
	S2-S4	PF-FW	0.430	7.91	0.093	.
	S3-S4	PF-FW	-0.344	5.05	0.255	
	S1-S2	PF-PM	-0.384	6.29	0.162	
	S1-S3	PF-PM	0.700	20.89	<0.001	***
	S1-S4	PF-PM	0.016	0.01	1	
	S2-S3	PF-PM	1.083	50.09	<0.001	***
	S2-S4	PF-PM	0.400	6.83	0.147	
	S3-S4	PF-PM	-0.683	19.94	0.001	***
	S1-S2	FW-PM	0.006	0.00	1	
	S1-S3	FW-PM	0.315	4.24	0.308	
	S1-S4	FW-PM	-0.025	0.03	1	
	S2-S3	FW-PM	0.309	4.08	0.308	
	S2-S4	FW-PM	-0.031	0.04	1	
	S3-S4	FW-PM	-0.340	4.92	0.255	
k) *Arg*	S1-S2	PF-FW	1.145	151.11	<0.001	***
	S1-S3	PF-FW	0.764	59.16	<0.001	***
	S1-S4	PF-FW	0.798	79.95	<0.001	***
	S2-S3	PF-FW	-0.381	24.84	<0.001	***
	S2-S4	PF-FW	-0.347	30.37	<0.001	***
	S3-S4	PF-FW	0.035	0.23	1	
	S1-S2	PF-PM	0.379	20.60	<0.001	***
	S1-S3	PF-PM	-0.079	0.65	1	
	S1-S4	PF-PM	-0.294	14.59	0.002	**
	S2-S3	PF-PM	-0.458	29.46	<0.001	***
	S2-S4	PF-PM	-0.673	129.53	<0.001	***
	S3-S4	PF-PM	-0.216	7.66	0.030	*
	S1-S2	FW-PM	-0.766	71.20	<0.001	***
	S1-S3	FW-PM	-0.843	72.01	<0.001	***
	S1-S4	FW-PM	-1.093	158.40	<0.001	***
	S2-S3	FW-PM	-0.076	0.88	1	
	S2-S4	FW-PM	-0.327	24.63	<0.001	***
	S3-S4	FW-PM	-0.250	10.52	0.010	**
l) *Cys*	S1-S2	PF-FW	0.006	0.04	1	
	S1-S3	PF-FW	-0.033	1.34	1	
	S1-S4	PF-FW	0.053	3.55	0.516	
	S2-S3	PF-FW	-0.039	1.85	0.897	
	S2-S4	PF-FW	0.048	2.83	0.685	
	S3-S4	PF-FW	0.086	9.24	0.035	*
	S1-S2	PF-PM	-0.095	11.25	0.015	*
	S1-S3	PF-PM	-0.138	23.71	<0.001	***
	S1-S4	PF-PM	0.070	6.04	0.152	
	S2-S3	PF-PM	-0.043	2.30	0.808	
	S2-S4	PF-PM	0.165	33.77	<0.001	***
	S3-S4	PF-PM	0.208	53.68	<0.001	***
	S1-S2	FW-PM	-0.101	12.64	0.009	**
	S1-S3	FW-PM	-0.105	13.78	0.006	**
	S1-S4	FW-PM	0.016	0.33	1	
	S2-S3	FW-PM	-0.004	0.02	1	
	S2-S4	FW-PM	0.117	17.05	0.002	**
	S3-S4	FW-PM	0.122	18.38	0.001	**

Table S9 continued.

AA		Strain	Diet	Value	*F*	*P*	
**m)**		S1-S2	PF-FW	1.106	21.48	<0.001	***
		S1-S3	PF-FW	0.954	17.73	0.001	**
		S1-S4	PF-FW	1.262	31.22	<0.001	***
		S2-S3	PF-FW	-0.152	0.93	1	
		S2-S4	PF-FW	0.156	1.00	1	
		S3-S4	PF-FW	0.307	5.05	0.198	
		S1-S2	PF-PM	-0.533	17.06	0.001	**
		S1-S3	PF-PM	-0.750	28.80	<0.001	***
	Glx (Glu + Gln)	S1-S4	PF-PM	-0.121	0.69	1	
		S2-S3	PF-PM	-0.217	2.03	0.957	
		S2-S4	PF-PM	0.413	6.89	0.099	.
		S3-S4	PF-PM	0.630	14.37	0.004	**
		S1-S2	FW-PM	-1.639	41.29	<0.001	***
		S1-S3	FW-PM	-1.705	60.13	<0.001	***
		S1-S4	FW-PM	-1.382	37.16	<0.001	***
		S2-S3	FW-PM	-0.065	0.14	1	
		S2-S4	FW-PM	0.257	1.92	0.957	
		S3-S4	FW-PM	0.322	5.87	0.147	
**n)**		S1-S2	PF-FW	-0.278	22.12	<0.001	***
		S1-S3	PF-FW	-0.259	17.35	0.002	**
		S1-S4	PF-FW	-0.464	58.32	<0.001	***
		S2-S3	PF-FW	0.019	0.15	1	
		S2-S4	PF-FW	-0.186	14.67	0.004	**
		S3-S4	PF-FW	-0.205	15.52	0.003	**
		S1-S2	PF-PM	-0.083	0.94	1	
		S1-S3	PF-PM	-0.074	0.64	1	
	Gly	S1-S4	PF-PM	-0.083	1.03	1	
		S2-S3	PF-PM	0.008	0.01	1	
		S2-S4	PF-PM	0.000	0.00	1	
		S3-S4	PF-PM	-0.009	0.01	1	
		S1-S2	FW-PM	0.196	4.30	0.424	
		S1-S3	FW-PM	0.185	3.59	0.566	
		S1-S4	FW-PM	0.381	18.16	0.001	**
		S2-S3	FW-PM	-0.011	0.02	1	
		S2-S4	FW-PM	0.185	6.74	0.138	
		S3-S4	FW-PM	0.197	6.80	0.138	
**o)**		S1-S2	PF-FW	-0.625	12.68	0.012	*
		S1-S3	PF-FW	-0.157	1.01	1	
		S1-S4	PF-FW	-0.705	8.64	0.070	.
		S2-S3	PF-FW	0.469	5.42	0.280	
		S2-S4	PF-FW	-0.080	0.09	1	
		S3-S4	PF-FW	-0.549	4.48	0.385	
		S1-S2	PF-PM	0.195	0.84	1	
		S1-S3	PF-PM	0.231	1.70	1	
	Pro	S1-S4	PF-PM	-0.517	5.14	0.297	
		S2-S3	PF-PM	0.037	0.03	1	
		S2-S4	PF-PM	-0.712	8.19	0.075	.
		S3-S4	PF-PM	-0.749	11.61	0.019	*
		S1-S2	FW-PM	0.820	14.64	0.006	**
		S1-S3	FW-PM	0.388	3.36	0.574	
		S1-S4	FW-PM	0.188	0.80	1	
		S2-S3	FW-PM	-0.432	4.01	0.448	
		S2-S4	FW-PM	-0.632	8.62	0.070	.
		S3-S4	FW-PM	-0.200	0.88	1	

AA		Strain	Diet	Value	*F*	*P*	
**p)**		S1-S2	PF-FW	0.166	1.25	1	
		S1-S3	PF-FW	0.187	1.57	1	
		S1-S4	PF-FW	0.351	5.56	0.390	
		S2-S3	PF-FW	0.020	0.02	1	
		S2-S4	PF-FW	0.184	1.54	1	
		S3-S4	PF-FW	0.164	1.22	1	
		S1-S2	PF-PM	0.051	0.12	1	
		S1-S3	PF-PM	0.019	0.02	1	
	Ser	S1-S4	PF-PM	0.073	0.24	1	
		S2-S3	PF-PM	-0.032	0.05	1	
		S2-S4	PF-PM	0.022	0.02	1	
		S3-S4	PF-PM	0.054	0.13	1	
		S1-S2	FW-PM	-0.115	0.60	1	
		S1-S3	FW-PM	-0.167	1.27	1	
		S1-S4	FW-PM	-0.278	3.48	1	
		S2-S3	FW-PM	-0.053	0.12	1	
		S2-S4	FW-PM	-0.163	1.20	1	
		S3-S4	FW-PM	-0.110	0.55	1	
**q)**		S1-S2	PF-FW	0.689	16.82	0.002	**
		S1-S3	PF-FW	0.063	0.12	1	
		S1-S4	PF-FW	0.470	8.08	0.067	.
		S2-S3	PF-FW	-0.626	16.48	0.002	**
		S2-S4	PF-FW	-0.219	2.81	0.692	
		S3-S4	PF-FW	0.407	7.24	0.092	.
		S1-S2	PF-PM	0.446	8.26	0.067	.
		S1-S3	PF-PM	0.021	0.02	1	
	Tyr	S1-S4	PF-PM	-0.079	0.23	1	
		S2-S3	PF-PM	-0.425	15.14	0.003	**
		S2-S4	PF-PM	-0.524	19.13	0.001	***
		S3-S4	PF-PM	-0.099	0.67	1	
		S1-S2	FW-PM	-0.243	5.12	0.218	
		S1-S3	FW-PM	-0.042	0.13	1	
		S1-S4	FW-PM	-0.548	37.69	<0.001	***
		S2-S3	FW-PM	0.201	2.13	0.899	
		S2-S4	FW-PM	-0.305	6.87	0.100	.
		S3-S4	FW-PM	-0.506	16.63	0.002	**

Table S10. Pairwise contrasts between strains within dietary levels for larval amino acid profiles (g/100 g protein).

AA: amino acid (according to conventional abbreviations). Pairwise strain-specific contrasts (S1-4) were performed within fixed dietary levels (poultry feed (PF), food waste (FW), poultry manure (PM)). Model-based estimates for all AA are reported on the response scale. *P*-values were adjusted for multiple simultaneous testing.

	AA	Diet	Strain-specific Contrast	Estimate	*P*
**a)**	*His*	PF	S1 – S2	-0.089	0.067
			S1 – S3	-0.165	0.001
			S1 – S4	-0.281	<0.001
			S2 – S3	-0.076	0.046
			S2 – S4	-0.191	<0.001
			S3 – S4	-0.115	0.050
		FW	S1 – S2	-0.087	0.018
			S1 – S3	-0.056	0.253
			S1 – S4	-0.193	<0.001
			S2 – S3	0.031	0.555
			S2 – S4	-0.106	0.001
			S3 – S4	-0.137	<0.001
		PM	S1 – S2	-0.235	0.001
			S1 – S3	-0.126	0.096
			S1 – S4	-0.195	0.002
			S2 – S3	0.109	0.061
			S2 – S4	0.039	0.753
			S3 – S4	-0.070	0.221
**b)**	*Ile*	PF	S1 – S2	0.044	0.928
			S1 – S3	0.006	1
			S1 – S4	-0.024	0.998
			S2 – S3	-0.037	0.900
			S2 – S4	-0.068	0.947
			S3 – S4	-0.030	0.996
		FW	S1 – S2	0.029	0.983
			S1 – S3	-0.060	0.823
			S1 – S4	0.053	0.910
			S2 – S3	-0.089	0.716
			S2 – S4	0.024	0.994
			S3 – S4	0.113	0.541
		PM	S1 – S2	0.017	0.997
			S1 – S3	-0.010	1
			S1 – S4	-0.164	0.411
			S2 – S3	-0.027	0.998
			S2 – S4	-0.181	0.143
			S3 – S4	-0.153	0.789
**c)**	*Leu*	PF	S1 – S2	0.128	0.029
			S1 – S3	0.071	0.261
			S1 – S4	-0.040	0.898
			S2 – S3	-0.057	0.759
			S2 – S4	-0.168	0.103
			S3 – S4	-0.111	0.373
		FW	S1 – S2	0.256	0.048
			S1 – S3	0.243	0.038
			S1 – S4	0.319	0.004
			S2 – S3	-0.013	0.995
			S2 – S4	0.063	0.674
			S3 – S4	0.076	0.248
		PM	S1 – S2	0.072	0.572
			S1 – S3	0.090	0.665
			S1 – S4	0.157	0.015
			S2 – S3	0.018	0.996
			S2 – S4	0.085	0.357
			S3 – S4	0.067	0.814

	AA	Diet	Strain-specific Contrast	Estimate	*P*
**d)**	*Lys*	PF	S1 – S2	-0.450	<0.001
			S1 – S3	-0.419	<0.001
			S1 – S4	-0.189	0.098
			S2 – S3	0.032	0.979
			S2 – S4	0.262	0.010
			S3 – S4	0.230	0.029
		FW	S1 – S2	-0.383	<0.001
			S1 – S3	0.188	0.100
			S1 – S4	0.175	0.141
			S2 – S3	0.571	<0.001
			S2 – S4	0.558	<0.001
			S3 – S4	-0.013	0.998
		PM	S1 – S2	-0.412	<0.001
			S1 – S3	-0.050	0.923
			S1 – S4	-0.123	0.426
			S2 – S3	0.362	<0.001
			S2 – S4	0.289	0.004
			S3 – S4	-0.073	0.802
**e)**	*Met*	PF	S1 – S2	-0.145	<0.001
			S1 – S3	-0.068	0.087
			S1 – S4	-0.085	0.227
			S2 – S3	0.077	0.011
			S2 – S4	0.060	0.474
			S3 – S4	-0.017	0.972
		FW	S1 – S2	0.130	<0.001
			S1 – S3	0.332	<0.001
			S1 – S4	0.185	0.001
			S2 – S3	0.202	<0.001
			S2 – S4	0.054	0.560
			S3 – S4	-0.148	0.029
		PM	S1 – S2	-0.089	0.146
			S1 – S3	-0.174	0.002
			S1 – S4	-0.205	0.001
			S2 – S3	-0.084	0.120
			S2 – S4	-0.115	0.040
			S3 – S4	-0.031	0.908
**f)**	*Phe*	PF	S1 – S2	-0.228	<0.001
			S1 – S3	0.014	0.984
			S1 – S4	0.028	0.929
			S2 – S3	0.242	<0.001
			S2 – S4	0.255	<0.001
			S3 – S4	0.014	0.993
		FW	S1 – S2	-0.013	0.997
			S1 – S3	0.152	0.073
			S1 – S4	0.157	0.036
			S2 – S3	0.166	0.006
			S2 – S4	0.170	0.001
			S3 – S4	0.004	0.999
		PM	S1 – S2	-0.077	0.320
			S1 – S3	-0.147	0.117
			S1 – S4	-0.172	0.011
			S2 – S3	-0.070	0.701
			S2 – S4	-0.095	0.292
			S3 – S4	-0.025	0.985

Table S10 continued.

	AA	Diet	Strain-specific Contrast	Estimate	*P*
**g)**	*Thr*	PF	S1 – S2	0.011	0.988
			S1 – S3	0.019	0.943
			S1 – S4	0.018	0.990
			S2 – S3	0.008	0.989
			S2 – S4	0.006	0.999
			S3 – S4	-0.002	1
		FW	S1 – S2	0.112	0.384
			S1 – S3	0.101	0.159
			S1 – S4	0.126	0.149
			S2 – S3	-0.011	0.997
			S2 – S4	0.013	0.997
			S3 – S4	0.024	0.895
		PM	S1 – S2	0.113	0.002
			S1 – S3	-0.069	0.591
			S1 – S4	-0.176	<0.001
			S2 – S3	-0.182	0.004
			S2 – S4	-0.289	<0.001
			S3 – S4	-0.107	0.242

	AA	Diet	Strain-specific Contrast	Estimate	*P*
**h)**	*Val*	PF	S1 – S2	-0.245	0.031
			S1 – S3	-0.489	<0.001
			S1 – S4	-0.361	0.001
			S2 – S3	-0.244	<0.001
			S2 – S4	-0.116	0.014
			S3 – S4	0.128	0.077
		FW	S1 – S2	0.323	0.084
			S1 – S3	0.052	0.953
			S1 – S4	-0.093	0.700
			S2 – S3	-0.271	0.239
			S2 – S4	-0.415	0.014
			S3 – S4	-0.145	0.464
		PM	S1 – S2	-0.252	0.276
			S1 – S3	-0.061	0.950
			S1 – S4	-0.146	0.574
			S2 – S3	0.191	0.415
			S2 – S4	0.106	0.821
			S3 – S4	-0.085	0.801

	AA	Diet	Strain-specific Contrast	Estimate	*P*
**i)**	*Ala*	PF	S1 – S2	0.535	0.004
			S1 – S3	1.064	<0.001
			S1 – S4	1.088	<0.001
			S2 – S3	0.529	<0.001
			S2 – S4	0.552	<0.001
			S3 – S4	0.024	0.974
		FW	S1 – S2	1.220	<0.001
			S1 – S3	1.219	<0.001
			S1 – S4	1.543	<0.001
			S2 – S3	-0.002	1
			S2 – S4	0.323	0.021
			S3 – S4	0.324	0.019
		PM	S1 – S2	0.508	0.001
			S1 – S3	0.602	0.002
			S1 – S4	0.382	0.012
			S2 – S3	0.094	0.913
			S2 – S4	-0.127	0.557
			S3 – S4	-0.220	0.361

	AA	Diet	Strain-specific Contrast	Estimate	*P*
**j)**	*Asx (Asp + Asn)*	PF	S1 – S2	0.028	0.994
			S1 – S3	0.419	0.002
			S1 – S4	0.425	0.001
			S2 – S3	0.391	0.003
			S2 – S4	0.397	0.003
			S3 – S4	0.006	1
		FW	S1 – S2	0.418	0.002
			S1 – S3	0.034	0.989
			S1 – S4	0.384	0.004
			S2 – S3	-0.384	0.004
			S2 – S4	-0.034	0.989
			S3 – S4	0.350	0.010
		PM	S1 – S2	0.412	0.002
			S1 – S3	-0.281	0.056
			S1 – S4	0.409	0.002
			S2 – S3	-0.693	<0.001
			S2 – S4	-0.003	1
			S3 – S4	0.690	<0.001

	AA	Diet	Strain-specific Contrast	Estimate	*P*
**k)**	*Arg*	PF	S1 – S2	0.026	0.974
			S1 – S3	-0.172	0.073
			S1 – S4	-0.251	<0.001
			S2 – S3	-0.197	0.005
			S2 – S4	-0.277	<0.001
			S3 – S4	-0.080	0.412
		FW	S1 – S2	-1.119	<0.001
			S1 – S3	-0.935	<0.001
			S1 – S4	-1.050	<0.001
			S2 – S3	0.184	0.004
			S2 – S4	0.070	0.492
			S3 – S4	-0.114	0.114
		PM	S1 – S2	-0.353	<0.001
			S1 – S3	-0.093	0.539
			S1 – S4	0.043	0.846
			S2 – S3	0.260	0.001
			S2 – S4	0.396	<0.001
			S3 – S4	0.136	0.106

	AA	Diet	Strain-specific Contrast	Estimate	*P*
**l)**	*Cys*	PF	S1 – S2	-0.022	0.690
			S1 – S3	-0.049	0.080
			S1 – S4	0.061	0.019
			S2 – S3	-0.027	0.540
			S2 – S4	0.083	0.001
			S3 – S4	0.110	<0.001
		FW	S1 – S2	-0.028	0.513
			S1 – S3	-0.016	0.851
			S1 – S4	0.007	0.984
			S2 – S3	0.012	0.938
			S2 – S4	0.035	0.310
			S3 – S4	0.023	0.650
		PM	S1 – S2	0.073	0.003
			S1 – S3	0.089	<0.001
			S1 – S4	-0.009	0.969
			S2 – S3	0.016	0.853
			S2 – S4	-0.082	0.001
			S3 – S4	-0.098	<0.001

Table S10 continued.

	AA	Diet	Strain-specific Contrast	Estimate	*P*
**m)**	*Glx (Glu + Gln)*	PF	S1 – S2	0.087	0.551
			S1 – S3	0.109	0.734
			S1 – S4	0.154	0.437
			S2 – S3	0.023	0.994
			S2 – S4	0.067	0.859
			S3 – S4	0.045	0.982
		FW	S1 – S2	-1.020	<0.001
			S1 – S3	-0.845	0.001
			S1 – S4	-1.108	<0.001
			S2 – S3	0.174	0.528
			S2 – S4	-0.089	0.904
			S3 – S4	-0.263	0.001
		PM	S1 – S2	0.620	<0.001
			S1 – S3	0.860	<0.001
			S1 – S4	0.274	0.050
			S2 – S3	0.240	0.215
			S2 – S4	-0.346	0.054
			S3 – S4	-0.585	<0.001
**n)**	*Gly*	PF	S1 – S2	0.154	<0.001
			S1 – S3	0.135	0.005
			S1 – S4	-0.045	0.577
			S2 – S3	-0.019	0.946
			S2 – S4	-0.199	<0.001
			S3 – S4	-0.180	<0.001
		FW	S1 – S2	0.433	<0.001
			S1 – S3	0.394	<0.001
			S1 – S4	0.420	<0.001
			S2 – S3	-0.039	0.711
			S2 – S4	-0.013	0.987
			S3 – S4	0.026	0.883
		PM	S1 – S2	0.237	0.021
			S1 – S3	0.209	0.074
			S1 – S4	0.039	0.954
			S2 – S3	-0.028	0.982
			S2 – S4	-0.198	0.009
			S3 – S4	-0.171	0.061
**o)**	*Pro*	PF	S1 – S2	0.003	1
			S1 – S3	0.070	0.783
			S1 – S4	-0.340	0.245
			S2 – S3	0.066	0.959
			S2 – S4	-0.344	0.372
			S3 – S4	-0.410	0.139
		FW	S1 – S2	0.629	<0.001
			S1 – S3	0.226	0.359
			S1 – S4	0.365	0.108
			S2 – S3	-0.402	0.048
			S2 – S4	-0.264	0.417
			S3 – S4	0.139	0.866
		PM	S1 – S2	-0.191	0.690
			S1 – S3	-0.161	0.749
			S1 – S4	0.177	0.587
			S2 – S3	0.030	0.997
			S2 – S4	0.368	0.033
			S3 – S4	0.338	0.022
**p)**	*Ser*	PF	S1 – S2	0.214	0.189
			S1 – S3	0.141	0.540
			S1 – S4	0.244	0.104
			S2 – S3	-0.072	0.901
			S2 – S4	0.031	0.991
			S3 – S4	0.103	0.760
		FW	S1 – S2	0.047	0.969
			S1 – S3	-0.045	0.973
			S1 – S4	-0.106	0.744
			S2 – S3	-0.093	0.814
			S2 – S4	-0.154	0.468
			S3 – S4	-0.061	0.938
		PM	S1 – S2	0.162	0.419
			S1 – S3	0.122	0.654
			S1 – S4	0.171	0.370
			S2 – S3	-0.040	0.981
			S2 – S4	0.009	1
			S3 – S4	0.049	0.966
**q)**	*Tyr*	PF	S1 – S2	-0.070	0.961
			S1 – S3	-0.657	<0.001
			S1 – S4	-0.430	0.031
			S2 – S3	-0.587	<0.001
			S2 – S4	-0.360	0.002
			S3 – S4	0.227	0.147
		FW	S1 – S2	-0.759	<0.001
			S1 – S3	-0.720	<0.001
			S1 – S4	-0.900	<0.001
			S2 – S3	0.039	0.989
			S2 – S4	-0.141	0.405
			S3 – S4	-0.180	0.355
		PM	S1 – S2	-0.516	<0.001
			S1 – S3	-0.678	<0.001
			S1 – S4	-0.352	<0.001
			S2 – S3	-0.162	0.041
			S2 – S4	0.164	0.128
			S3 – S4	0.326	<0.001

Table S11. Pairwise contrasts between diets within strains for larval amino acid profiles (g/100 g protein).

AA: amino acid (according to conventional abbreviations). Pairwise diet-specific contrasts (poultry feed (PF), food waste (FW) and poultry manure (PM)) were performed within fixed levels of the factor strain (S1-4). Model-based estimates for all AA are reported on the response scale. *P*-values were adjusted for multiple simultaneous testing.

	AA	Strain	Diet-specific Contrast	Estimate	*P*
**a)**	*His*	S1	PF – FW	-0.007	0.983
			PF – PM	0.465	<0.001
			FW – PM	0.472	<0.001
		S2	PF – FW	-0.005	0.965
			PF – PM	0.319	<0.001
			FW – PM	0.325	<0.001
		S3	PF – FW	0.102	0.004
			PF – PM	0.504	<0.001
			FW – PM	0.403	<0.001
		S4	PF – FW	0.080	0.152
			PF – PM	0.550	<0.001
			FW – PM	0.470	<0.001
**b)**	*Ile*	S1	PF – FW	0.047	0.834
			PF – PM	0.000	1
			FW – PM	-0.047	0.846
		S2	PF – FW	0.033	0.881
			PF – PM	-0.027	0.768
			FW – PM	-0.060	0.701
		S3	PF – FW	-0.019	0.965
			PF – PM	-0.017	0.994
			FW – PM	0.002	1
		S4	PF – FW	0.124	0.645
			PF – PM	-0.140	0.598
			FW – PM	-0.264	0.029
**c)**	*Leu*	S1	PF – FW	-0.214	0.037
			PF – PM	0.022	0.847
			FW – PM	0.236	0.035
		S2	PF – FW	-0.086	0.385
			PF – PM	-0.034	0.833
			FW – PM	0.051	0.684
		S3	PF – FW	-0.042	0.642
			PF – PM	0.041	0.861
			FW – PM	0.082	0.506
		S4	PF – FW	0.145	0.071
			PF – PM	0.219	0.004
			FW – PM	0.073	0.226
**d)**	*Lys*	S1	PF – FW	-0.188	0.058
			PF – PM	-0.729	<0.001
			FW – PM	-0.541	<0.001
		S2	PF – FW	-0.121	0.298
			PF – PM	-0.691	<0.001
			FW – PM	-0.57	<0.001
		S3	PF – FW	0.419	<0.001
			PF – PM	-0.361	<0.001
			FW – PM	-0.78	<0.001
		S4	PF – FW	0.176	0.081
			PF – PM	-0.663	<0.001
			FW – PM	-0.839	<0.001

	AA	Strain	Diet-specific Contrast	Estimate	*P*
**e)**	*Met*	S1	PF – FW	-0.235	<0.001
			PF – PM	0.101	0.053
			FW – PM	0.336	<0.001
		S2	PF – FW	0.040	0.176
			PF – PM	0.157	<0.001
			FW – PM	0.116	<0.001
		S3	PF – FW	0.165	<0.001
			PF – PM	-0.005	0.988
			FW – PM	-0.170	0.001
		S4	PF – FW	0.035	0.797
			PF – PM	-0.019	0.928
			FW – PM	-0.054	0.577
**f)**	*Phe*	S1	PF – FW	0.047	0.720
			PF – PM	0.231	<0.001
			FW – PM	0.185	0.013
		S2	PF – FW	0.261	<0.001
			PF – PM	0.382	<0.001
			FW – PM	0.121	0.049
		S3	PF – FW	0.184	<0.001
			PF – PM	0.070	0.526
			FW – PM	-0.115	0.172
		S4	PF – FW	0.175	<0.001
			PF – PM	0.031	0.850
			FW – PM	-0.144	0.007
**g)**	*Thr*	S1	PF – FW	-0.224	0.001
			PF – PM	-0.007	0.983
			FW – PM	0.217	<0.001
		S2	PF – FW	-0.123	0.071
			PF – PM	0.095	0.001
			FW – PM	0.217	0.001
		S3	PF – FW	-0.142	<0.001
			PF – PM	-0.095	0.167
			FW – PM	0.047	0.614
		S4	PF – FW	-0.116	0.130
			PF – PM	-0.201	0.002
			FW – PM	-0.085	0.143
**h)**	*Val*	S1	PF – FW	-0.230	0.071
			PF – PM	0.014	0.993
			FW – PM	0.244	0.078
		S2	PF – FW	0.338	0.020
			PF – PM	0.008	0.997
			FW – PM	-0.330	0.098
		S3	PF – FW	0.312	0.003
			PF – PM	0.442	<0.001
			FW – PM	0.131	0.419
		S4	PF – FW	0.039	0.823
			PF – PM	0.230	0.005
			FW – PM	0.191	0.086

Table S11 continued.

	AA	Strain	Diet-specific Contrast	Estimate	*P*
**i)**	*Ala*	S1	PF – FW	-1.051	<0.001
			PF – PM	0.321	0.175
			FW – PM	1.373	<0.001
		S2	PF – FW	-0.367	0.001
			PF – PM	0.294	0.005
			FW – PM	0.661	<0.001
		S3	PF – FW	-0.897	<0.001
			PF – PM	-0.141	0.501
			FW – PM	0.756	<0.001
		S4	PF – FW	-0.596	<0.001
			PF – PM	-0.385	<0.001
			FW – PM	0.211	0.056
**j)**	*Arg*	S1	PF – FW	0.744	<0.001
			PF – PM	0.583	<0.001
			FW – PM	-0.161	0.093
		S2	PF – FW	-0.401	<0.001
			PF – PM	0.204	<0.001
			FW – PM	0.605	<0.001
		S3	PF – FW	-0.020	0.941
			PF – PM	0.662	<0.001
			FW – PM	0.682	<0.001
		S4	PF – FW	-0.054	0.377
			PF – PM	0.878	<0.001
			FW – PM	0.932	<0.001
**k)**	*Asx*	S1	PF – FW	0.110	0.570
			PF – PM	0.168	0.275
			FW – PM	0.058	0.855
		S2	PF – FW	0.500	<0.001
			PF – PM	0.552	<0.001
			FW – PM	0.052	0.881
		S3	PF – FW	-0.275	0.036
			PF – PM	-0.532	<0.001
			FW – PM	-0.257	0.053
		S4	PF – FW	0.069	0.799
			PF – PM	0.152	0.347
			FW – PM	0.082	0.728
**l)**	*Cys*	S1	PF – FW	-0.019	0.618
			PF – PM	-0.407	<0.001
			FW – PM	-0.388	<0.001
		S2	PF – FW	-0.025	0.444
			PF – PM	-0.312	<0.001
			FW – PM	-0.288	<0.001
		S3	PF – FW	0.014	0.766
			PF – PM	-0.269	<0.001
			FW – PM	-0.283	<0.001
		S4	PF – FW	-0.072	0.002
			PF – PM	-0.477	<0.001
			FW – PM	-0.405	<0.001
**m)**	*Glx*	S1	PF – FW	0.949	<0.001
			PF – PM	-2.116	<0.001
			FW – PM	-3.066	<0.001
		S2	PF – FW	-0.157	0.414
			PF – PM	-1.583	<0.001
			FW – PM	-1.426	<0.001
		S3	PF – FW	-0.005	0.999
			PF – PM	-1.366	<0.001
			FW – PM	-1.361	<0.001
		S4	PF – FW	-0.313	0.005
			PF – PM	-1.996	<0.001
			FW – PM	-1.683	<0.001

	AA	Strain	Diet-specific Contrast	Estimate	*P*
**n)**	*Gly*	S1	PF – FW	-0.327	<0.001
			PF – PM	-0.358	<0.001
			FW – PM	-0.031	0.915
		S2	PF – FW	-0.049	0.303
			PF – PM	-0.276	<0.001
			FW – PM	-0.227	<0.001
		S3	PF – FW	-0.068	0.178
			PF – PM	-0.284	<0.001
			FW – PM	-0.216	0.002
		S4	PF – FW	0.137	0.001
			PF – PM	-0.275	<0.001
			FW – PM	-0.412	<0.001
**o)**	*Pro*	S1	PF – FW	-0.768	<0.001
			PF – PM	0.449	0.004
			FW – PM	1.216	<0.001
		S2	PF – FW	-0.142	0.625
			PF – PM	0.254	0.284
			FW – PM	0.396	0.034
		S3	PF – FW	-0.611	<0.001
			PF – PM	0.217	0.164
			FW – PM	0.828	<0.001
		S4	PF – FW	-0.062	0.958
			PF – PM	0.966	<0.001
			FW – PM	1.028	<0.001
**p)**	*Ser*	S1	PF – FW	-0.074	0.745
			PF – PM	-0.115	0.535
			FW – PM	-0.042	0.895
		S2	PF – FW	-0.240	0.026
			PF – PM	-0.167	0.008
			FW – PM	0.073	0.733
		S3	PF – FW	-0.261	0.001
			PF – PM	-0.135	0.442
			FW – PM	0.126	0.571
		S4	PF – FW	-0.425	0.004
			PF – PM	-0.189	0.353
			FW – PM	0.236	0.140
**q)**	*Tyr*	S1	PF – FW	1.176	<0.001
			PF – PM	1.243	<0.001
			FW – PM	0.068	0.433
		S2	PF – FW	0.487	<0.001
			PF – PM	0.797	<0.001
			FW – PM	0.311	0.004
		S3	PF – FW	1.113	<0.001
			PF – PM	1.223	<0.001
			FW – PM	0.110	0.533
		S4	PF – FW	0.706	<0.001
			PF – PM	1.322	<0.001
			FW – PM	0.616	<0.001

Table S12. Specific protein concentration and nitrogen-to-protein conversion: Twofold interaction contrasts of pairwise strain and diet level comparisons, pairwise contrasts between strains within dietary levels, and pairwise contrasts between diets within strains.

Pairwise level combinations are indicated for the two factors strain (S1-4) and diet (poultry feed (PF), food waste (FW), poultry manure (PM)). Interaction contrasts (panels a and d) are based on one degree of freedom (DF) on 60 residual DFs throughout. Estimates for pairwise contrasts of a given factor within fixed levels of another (panels b, c, e and f) are reported on the response scales. *P*-values were adjusted for multiple simultaneous testing.

a) Specific protein concentration (g/kg DM)

Strain	Diet	Value	*F*	*P*	
S1-S2	PF-FW	18.39	10.86	0.018	*
S1-S3	PF-FW	15.92	7.89	0.047	*
S1-S4	PF-FW	30.61	33.87	<0.001	***
S2-S3	PF-FW	-2.47	0.28	1	
S2-S4	PF-FW	12.22	8.73	0.040	*
S3-S4	PF-FW	14.69	11.97	0.013	*
S1-S2	PF-PM	1.93	0.14	1	
S1-S3	PF-PM	17.86	12.21	0.013	*
S1-S4	PF-PM	-1.33	0.09	1	
S2-S3	PF-PM	15.93	8.16	0.047	*
S2-S4	PF-PM	-3.25	0.44	1	
S3-S4	PF-PM	-19.19	15.42	0.003	**
S1-S2	FW-PM	-16.46	6.97	0.063	.
S1-S3	FW-PM	1.95	0.10	1	
S1-S4	FW-PM	-31.93	31.33	<0.001	***
S2-S3	FW-PM	18.41	11.03	0.018	*
S2-S4	FW-PM	-15.47	9.26	0.035	*
S3-S4	FW-PM	-33.88	47.06	<0.001	***

d) *Kp* (g protein/g nitrogen)

Strain	Diet	Value	*F*	*P*
S1-S2	PF-FW	0.148	2.619	1
S1-S3	PF-FW	0.104	1.223	1
S1-S4	PF-FW	0.144	2.656	1
S2-S3	PF-FW	-0.045	0.500	1
S2-S4	PF-FW	-0.004	0.007	1
S3-S4	PF-FW	0.040	0.479	1
S1-S2	PF-PM	0.229	4.890	0.432
S1-S3	PF-PM	0.114	3.511	0.856
S1-S4	PF-PM	-0.011	0.034	1
S2-S3	PF-PM	-0.115	1.441	1
S2-S4	PF-PM	-0.241	6.112	0.261
S3-S4	PF-PM	-0.125	6.471	0.231
S1-S2	FW-PM	0.081	0.427	1
S1-S3	FW-PM	0.010	0.011	1
S1-S4	FW-PM	-0.155	2.722	1
S2-S3	FW-PM	-0.071	0.474	1
S2-S4	FW-PM	-0.236	5.274	0.377
S3-S4	FW-PM	-0.165	6.591	0.230

b) Specific protein concentration (g/kg DM)

Diet	Strain-specific Contrast	Estimate	*P*
PF	S1 – S2	-21.54	<0.001
	S1 – S3	-11.18	0.005
	S1 – S4	-13.06	<0.001
	S2 – S3	10.36	0.014
	S2 – S4	8.48	0.016
	S3 – S4	-1.88	0.920
FW	S1 – S2	-39.93	<0.001
	S1 – S3	-27.09	<0.001
	S1 – S4	-43.67	<0.001
	S2 – S3	12.84	0.001
	S2 – S4	-3.74	0.623
	S3 – S4	-16.57	<0.001
PM	S1 – S2	-23.47	<0.001
	S1 – S3	-29.04	<0.001
	S1 – S4	-11.73	0.006
	S2 – S3	-5.57	0.604
	S2 – S4	11.74	0.026
	S3 – S4	17.31	<0.001

e) *Kp* (g protein/g nitrogen)

Diet	Strain-specific Contrast	Estimate	*P*
PF	S1 – S2	0.021	0.966
	S1 – S3	0.003	1
	S1 – S4	0.084	0.128
	S2 – S3	-0.018	0.962
	S2 – S4	0.063	0.172
	S3 – S4	0.081	0.031
FW	S1 – S2	-0.128	0.393
	S1 – S3	-0.101	0.624
	S1 – S4	-0.060	0.876
	S2 – S3	0.027	0.954
	S2 – S4	0.068	0.441
	S3 – S4	0.041	0.848
PM	S1 – S2	-0.209	0.129
	S1 – S3	-0.111	0.060
	S1 – S4	0.095	0.235
	S2 – S3	0.098	0.692
	S2 – S4	0.304	0.009
	S3 – S4	0.206	<0.001

c) Specific protein concentration (g/kg DM)

Strain	Diet-specific Contrast	Estimate	*P*
S1	PF – FW	51.33	<0.001
	PF – PM	2.34	0.753
	FW – PM	-49.00	<0.001
S2	PF – FW	32.95	<0.001
	PF – PM	0.41	0.994
	FW – PM	-32.54	<0.001
S3	PF – FW	35.42	<0.001
	PF – PM	-15.53	0.001
	FW – PM	-50.94	<0.001
S4	PF – FW	20.73	<0.001
	PF – PM	3.66	0.416
	FW – PM	-17.06	<0.001

f) *Kp* (g protein/g nitrogen)

Strain	Diet-specific Contrast	Estimate	*P*
S1	PF – FW	0.241	0.012
	PF – PM	0.628	<0.001
	FW – PM	0.387	<0.001
S2	PF – FW	0.093	0.080
	PF – PM	0.399	<0.001
	FW – PM	0.307	0.004
S3	PF – FW	0.137	0.013
	PF – PM	0.515	<0.001
	FW – PM	0.377	<0.001
S4	PF – FW	0.097	0.017
	PF – PM	0.640	<0.001
	FW – PM	0.543	<0.001

Figure S1. Mitochondrial COI haplotype relatedness among experimental black soldier fly strains.

Unrooted neighbour-joining dendrogram of the mitochondrial COI sequences detected across the four experimental black soldier fly strains (S1-4), for further details see Table S2. Computations are based on uncorrected p-distances across 658 base pairs of the partial COI gene.

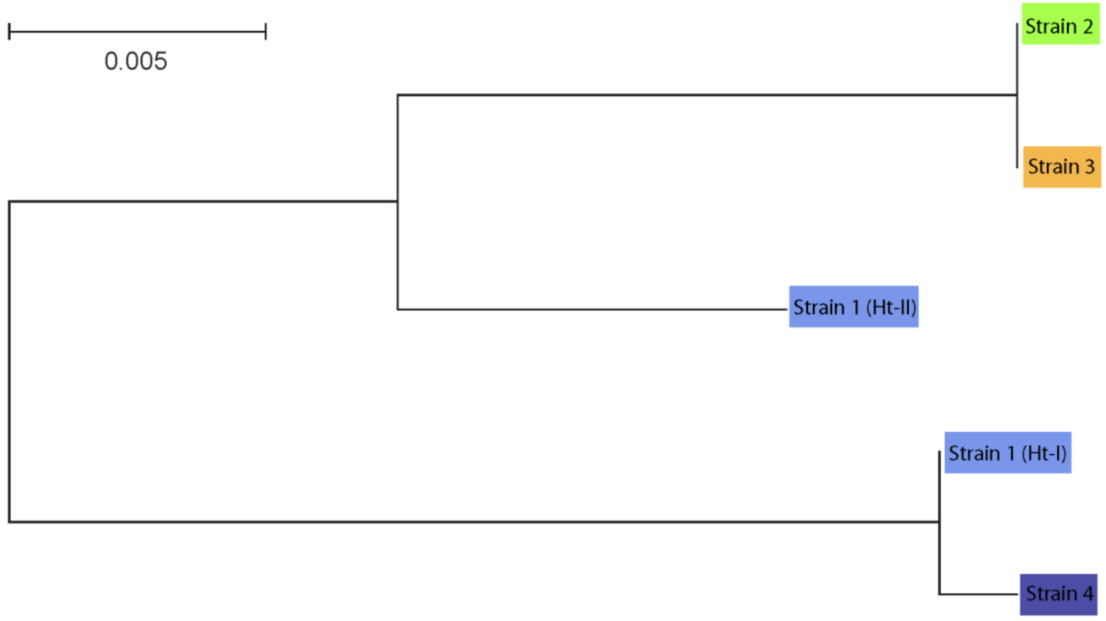

Figure S2. Larval weights at harvest.

Boxplots correspond to six replicates within each strain × diet combination. Standard errors of the means (cf. Table 3): Poultry feed: 2.9 (S1), 0.7 (S2), 1.6 (S3), 2.5 (S4); Food waste: 1.7 (S1), 1.8 (S2), 1.9 (S3), 1.4 (S4); Poultry manure: 0.9 (S1), 1.2 (S2), 0.7 (S3), 0.6 (S4).

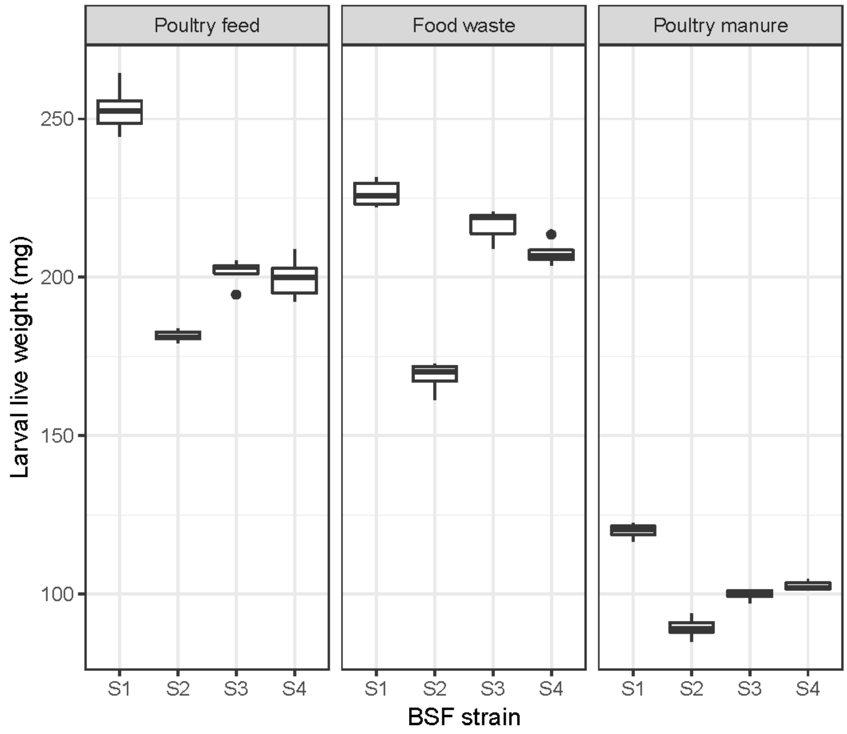

Figure S3. Larval mortality.

Boxplots correspond to six replicates within each strain × diet combination. Standard errors of the means (cf. Table 3): Poultry feed: 2.2 (S1), 0.8 (S2), 0.4 (S3), 0.4 (S4); Food waste: 0.5 (S1), 0.4 (S2), 0.1 (S3), 0.3 (S4); Poultry manure: 0.2 (S1), 0.3 (S2), 0.3 (S3), 0.2 (S4).

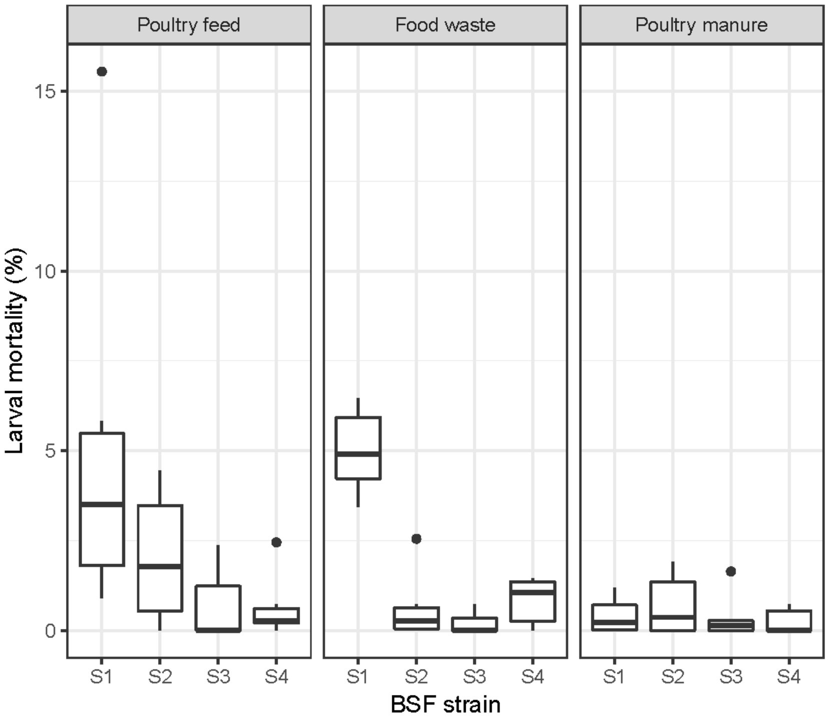

Figure S4. Larval biomass production.

Boxplots correspond to six replicates within each strain × diet combination. Standard errors of the means (cf. Table 3): Poultry feed: 3.1 (S1), 1.5 (S2), 0.7 (S3), 0.7 (S4); Food waste: 1.9 (S1), 0.4 (S2), 0.6 (S3), 0.6 (S4); Poultry manure: 0.6 (S1), 0.4 (S2), 0.2 (S3), 0.2 (S4).

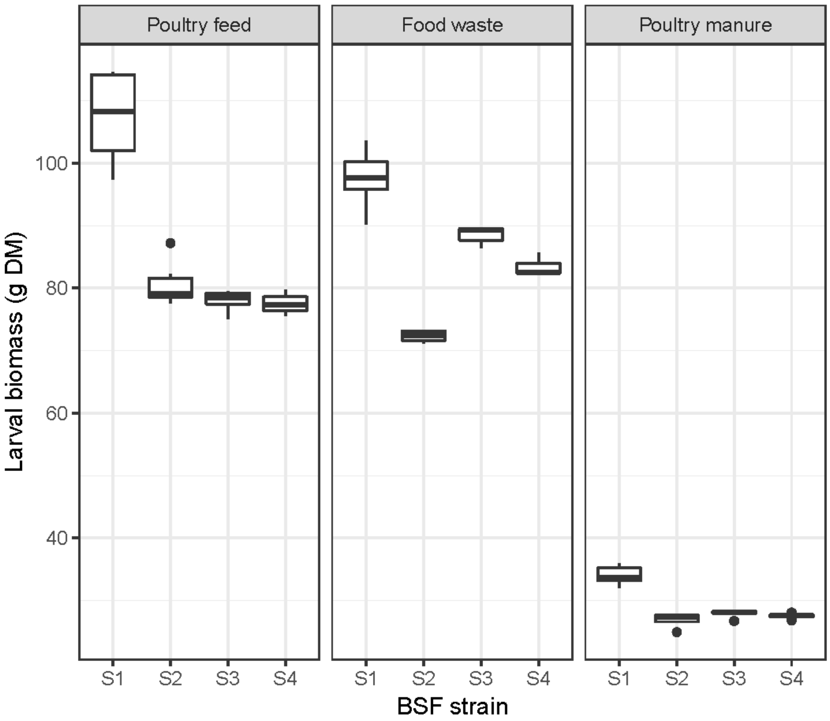

Figure S5. Larval estimated protein concentrations.

Boxplots correspond to six replicates within each strain × diet combination. Dry matter (DM) based concentrations of larval estimated protein refer to the nitrogen-to-protein conversion factor 4.76×N. Standard errors of the means (cf. Table 3): Poultry feed: 1.3 (S1), 1.5 (S2), 1.7 (S3), 1.5 (S4); Food waste: 0.9 (S1), 0.6 (S2), 1.6 (S3), 2.6 (S4); Poultry manure: 1.0 (S1), 6.5 (S2), 2.1 (S3), 3.7 (S4).

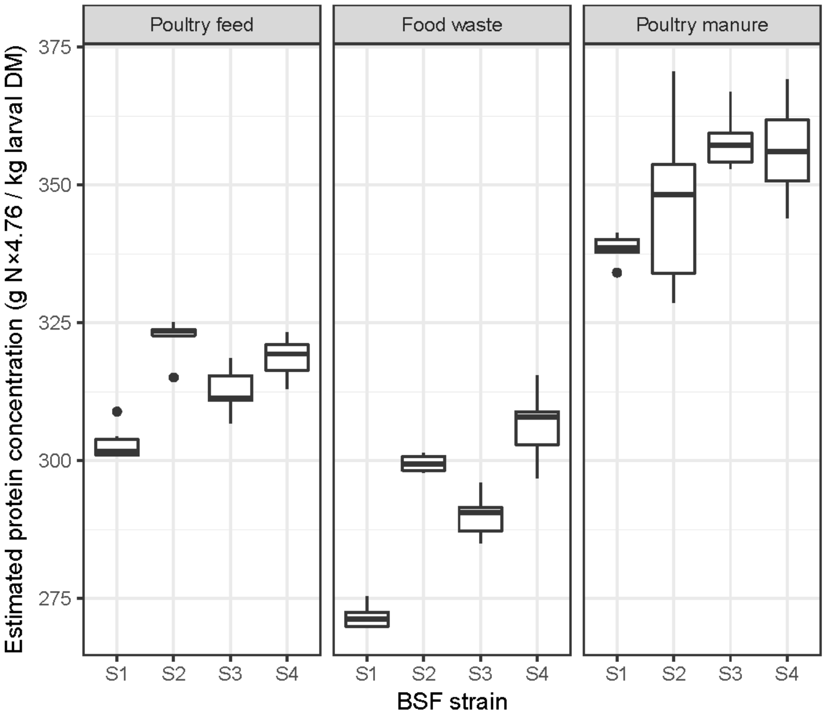

Figure S6. Total amounts of larval estimated protein.

Boxplots correspond to six replicates within each strain × diet combination. Productivity of total amounts of larval estimated protein refers to the nitrogen-to-protein conversion factor 4.76×N. Standard errors of the means (cf. Table 3): Poultry feed: 1.0 (S1), 0.4 (S2), 0.2 (S3), 0.2 (S4); Food waste: 0.5 (S1), 0.1 (S2), 0.3 (S3), 0.1 (S4); Poultry manure: 0.2 (S1), 0.2 (S2), 0.0 (S3), 0.1 (S4).

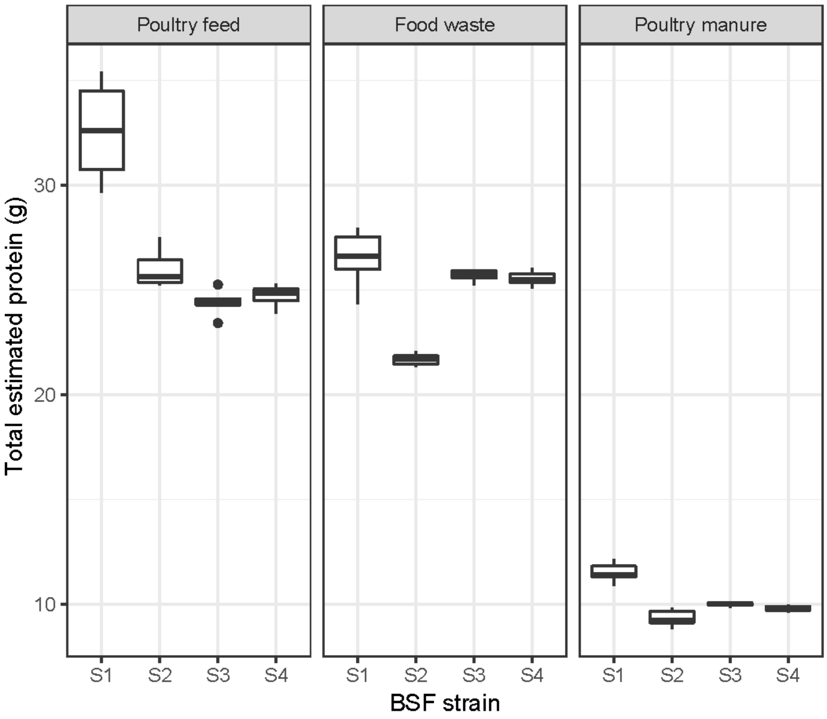

Figure S7. Larval ether extract concentrations.

Boxplots correspond to six replicates within each strain × diet combination. Calculations are dry matter (DM) based. Standard errors of the means (cf. Table 3): Poultry feed: 29.3 (S1), 17.5 (S2), 3.9 (S3), 5.7 (S4); Food waste: 4.5 (S1), 14.6 (S2), 14.6 (S3), 4.7 (S4); Poultry manure: 3.8 (S1), 22.1 (S2), 2.5 (S3), 3.2 (S4).

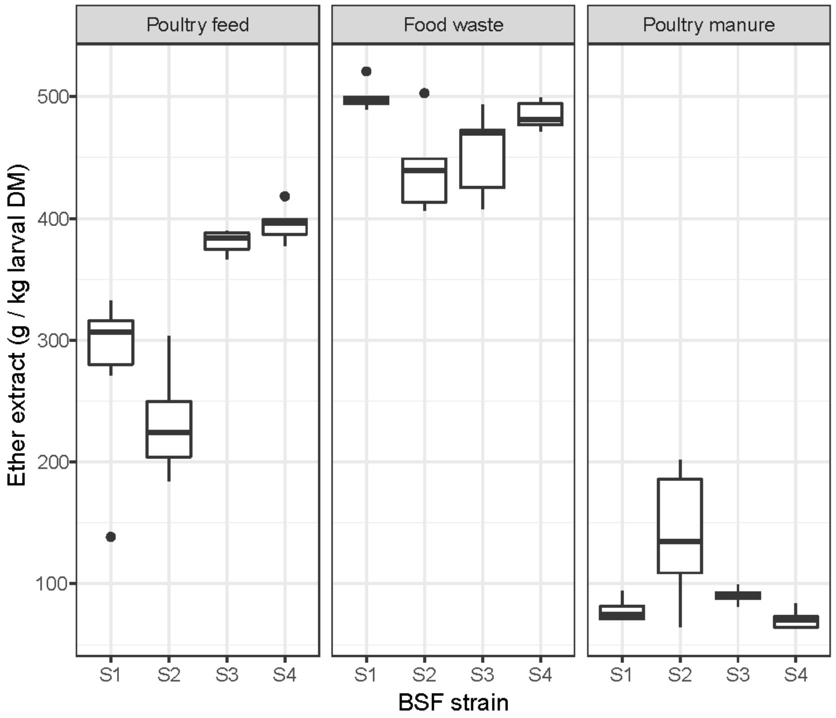

Figure S8. Total amounts of larval ether extract.

Boxplots correspond to six replicates within each strain × diet combination. Standard errors of the means (cf. Table 3): Poultry feed: 2.9 (S1), 1.4 (S2), 0.2 (S3), 0.5 (S4); Food waste: 1.1 (S1), 1.2 (S2), 1.2 (S3), 0.6 (S4); Poultry manure: 0.1 (S1), 0.6 (S2), 0.1 (S3), 0.1 (S4).

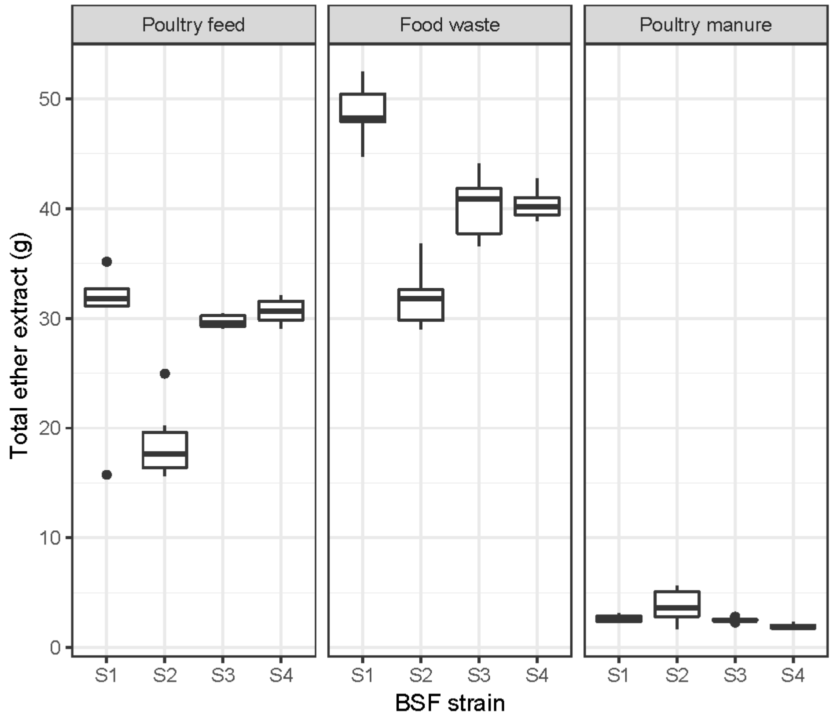

Figure S9. Ratios of larval estimated protein to ether extract.

Boxplots correspond to six replicates within each strain × diet combination. Calculations are based on total dry matter (DM). Standard errors of the means (cf. Table 3): Poultry feed: 0.2 (S1), 0.1 (S2), 0.01 (S3), 0.01 (S4); Food waste: 0.0 (S1), 0.02 (S2), 0.02 (S3), 0.01 (S4); Poultry manure: 0.2 (S1), 0.63 (S2), 0.1 (S3), 0.26 (S4).

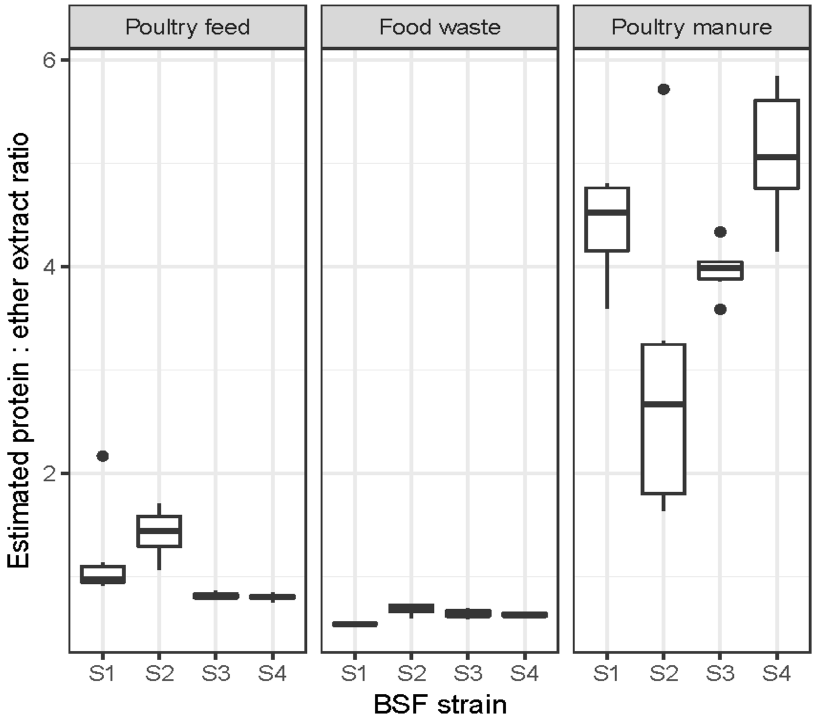

Figure S10. Larval total ash concentrations.

Boxplots correspond to six replicates within each strain × diet combination. Standard errors of the means (cf. Table 3): Poultry feed: 2.4 (S1), 1.4 (S2), 1.4 (S3), 0.9 (S4); Food waste: 0.3 (S1), 0.3 (S2), 0.2 (S3), 0.2 (S4); Poultry manure: 1.7 (S1), 2.1 (S2), 1.1 (S3), 1.4 (S4).

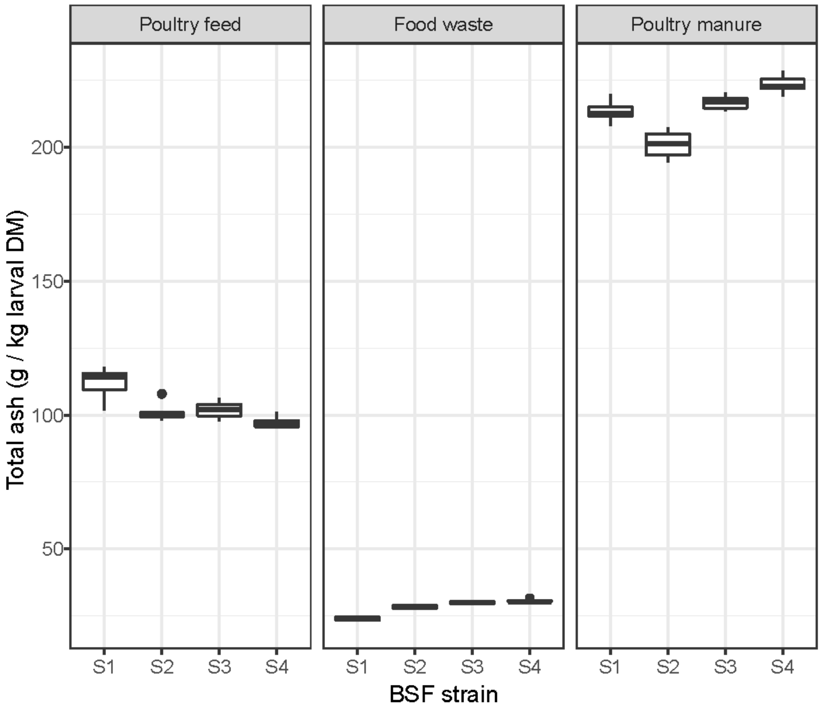

Figure S11. Amounts of larval total ash.

Boxplots correspond to six replicates within each strain × diet combination. Standard errors of the means (cf. Table 3): Poultry feed: 0.3 (S1), 0.3 (S2), 0.2 (S3), 0.1 (S4); Food waste: 0.1 (S1), 0.0 (S2), 0.0 (S3), 0.0 (S4); Poultry manure: 0.1 (S1), 0.1 (S2), 0.0 (S3), 0.1 (S4).

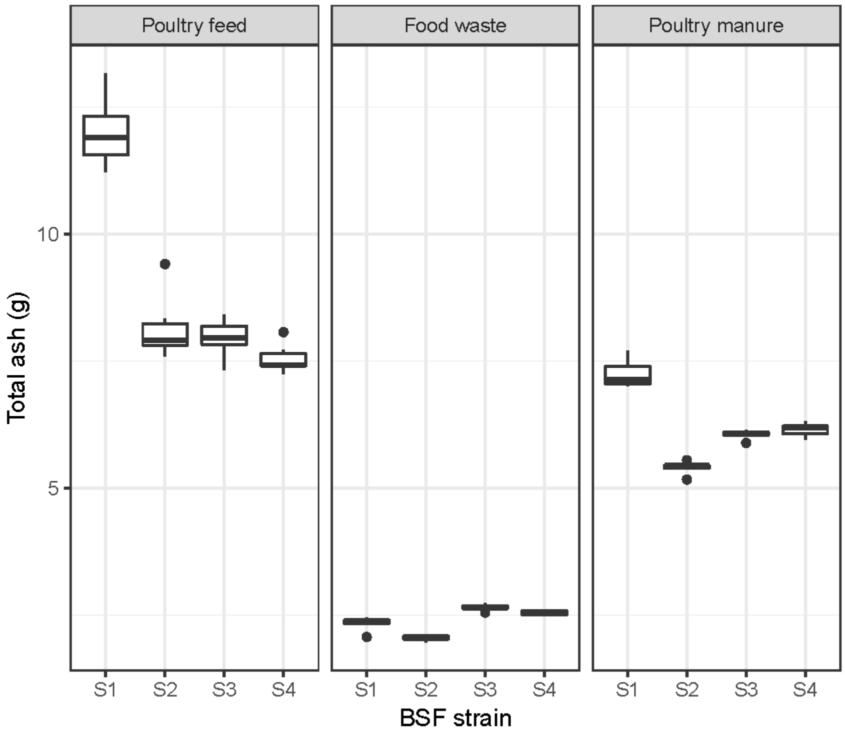

Figure S12. Larval bioconversion efficiency.

Boxplots correspond to six replicates within each strain × diet combination. Calculations are dry matter (DM) based. Standard errors of the means (cf. Table 3): Poultry feed: 10.0 (S1), 4.7 (S2), 2.2 (S3), 2.1 (S4); Food waste: 4.3 (S1), 0.9 (S2), 1.3 (S3), 1.3 (S4); Poultry manure: 1.5 (S1), 1.2 (S2), 0.6 (S3), 0.4 (S4).

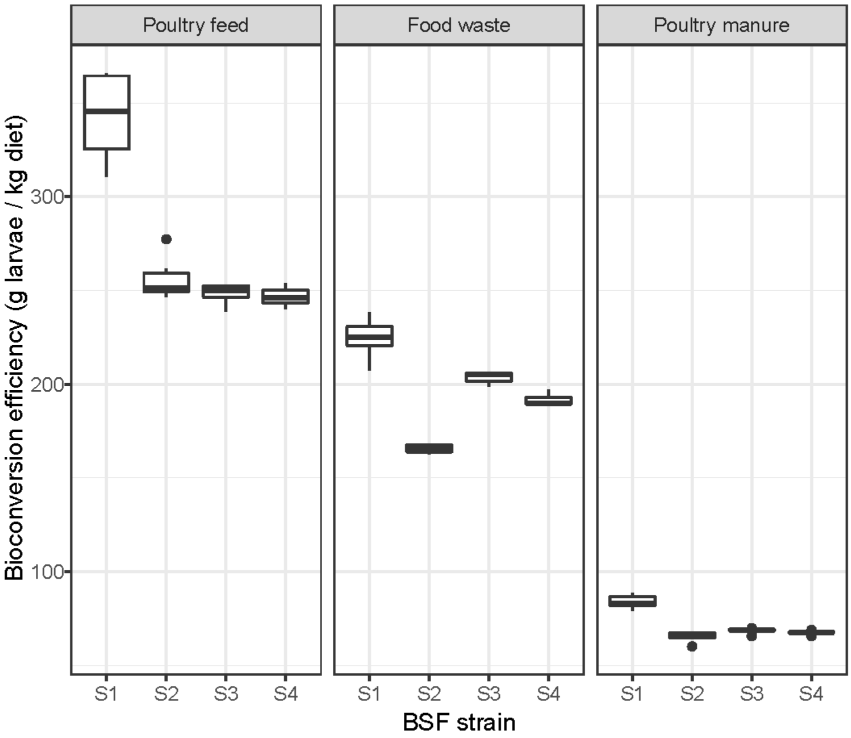

Figure S13. Larval nitrogen bioconversion efficiency.

Boxplots correspond to six replicates within each strain × diet combination. Standard errors of the means (cf. Table 3): Poultry feed: 20.7 (S1), 7.6 (S2), 5.2 (S3), 4.4 (S4); Food waste: 10.8 (S1), 2.4 (S2), 2.2 (S3), 2.8 (S4); Poultry manure: 2.1 (S1), 1.9 (S2), 0.5 (S3), 0.7 (S4).

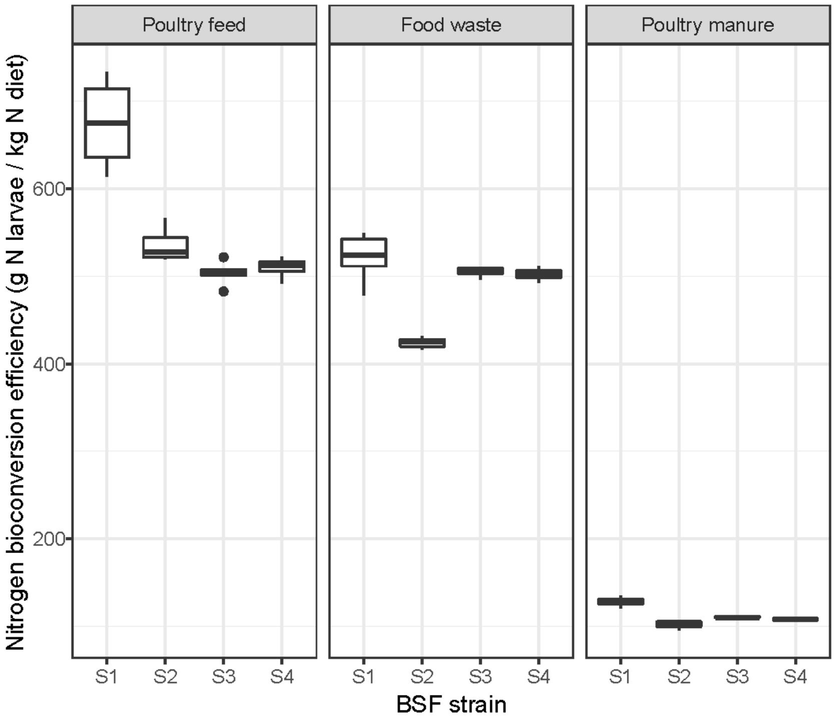

Figure S14. Reduction of dietary dry matter.

Boxplots correspond to six replicates within each strain × diet combination. This response refers to the reduction potential of organic waste. Standard errors of the means (cf. Table 3): Poultry feed: 9.6 (S1), 11.5 (S2), 5.4 (S3), 6.9 (S4); Food waste: 3.0 (S1), 17.2 (S2), 1.0 (S3), 4.8 (S4); Poultry manure: 2.2 (S1), 3.9 (S2), 2.1 (S3), 2.6 (S4).

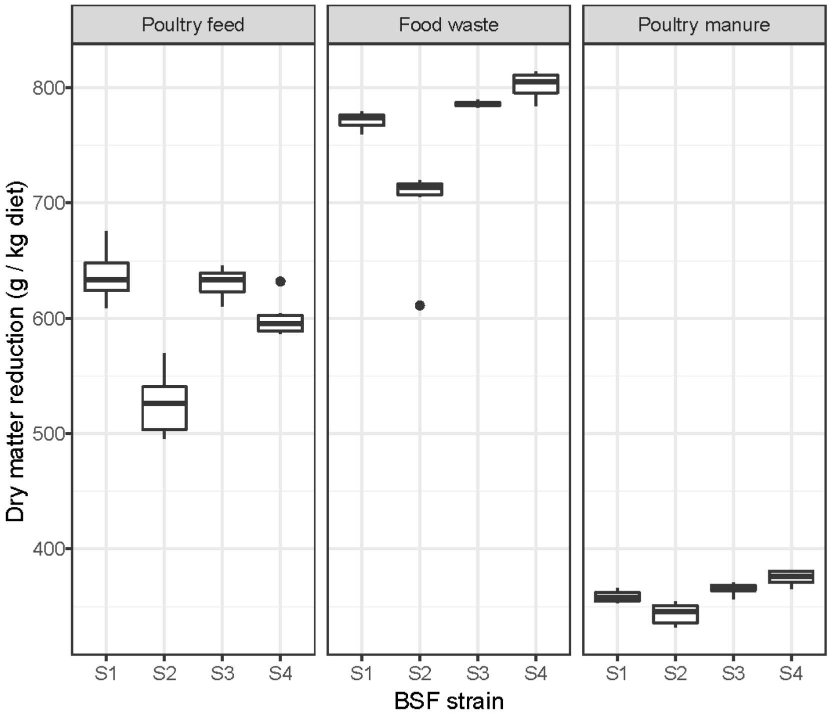

Figure S15. Reduction of dietary neutral detergent fibre.

Boxplots correspond to six replicates within each strain × diet combination. Standard errors of the means (cf. Table 3): Poultry feed: 16.1 (S1), 7.2 (S2), 25.4 (S3), 9.6 (S4); Food waste: 4.5 (S1), 9.7 (S2), 5.3 (S3), 15.7 (S4); Poultry manure: 17.5 (S1), 14.7 (S2), 17.0 (S3), 9.5 (S4).

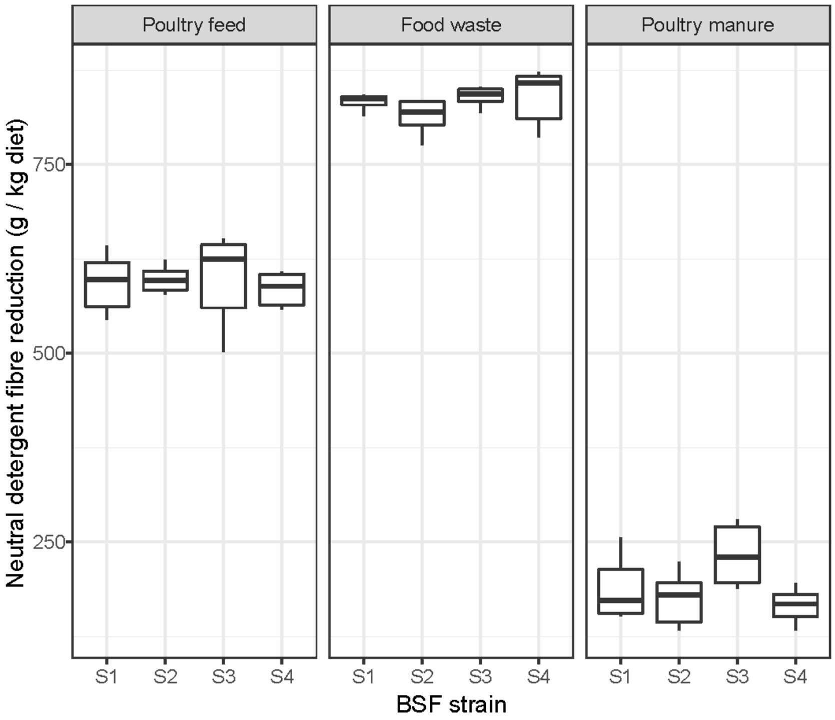

Figure S16. Reduction of dietary acid detergent fibre.

Boxplots correspond to six replicates within each strain × diet combination. Standard errors of the means (cf. Table 3): Poultry feed: 24.2 (S1), 15.0 (S2), 31.3 (S3), 11.2 (S4); Food waste: 12.9 (S1), 28.2 (S2), 13.8 (S3), 37.4 (S4); Poultry manure: 19.9 (S1), 17.9 (S2), 33.3 (S3), 10.6 (S4).

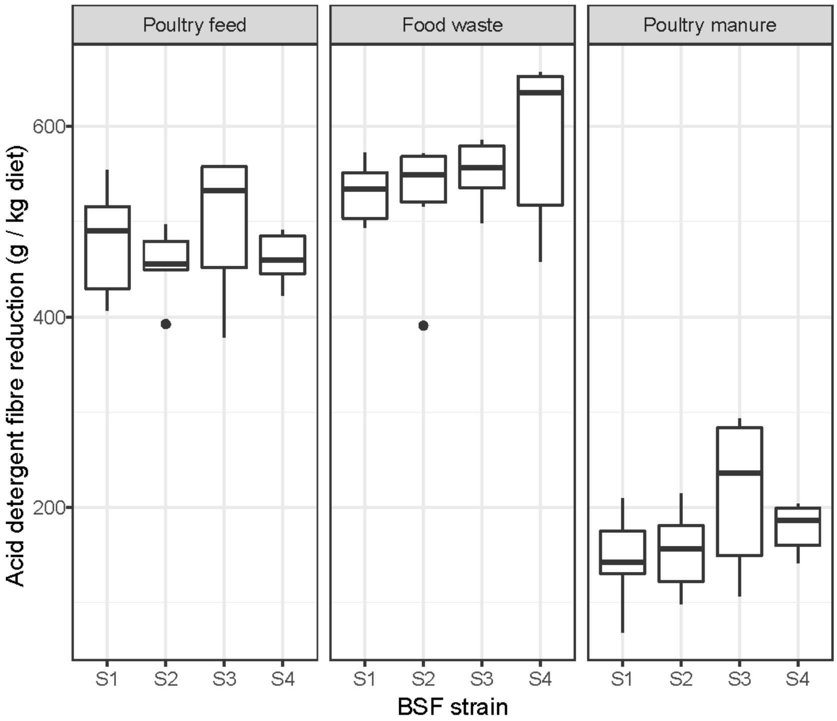

Figure S17. Reduction of dietary hemicellulose.

Boxplots correspond to six replicates within each strain × diet combination. Standard errors of the means (cf. Table 3): Poultry feed: 9.9 (S1), 14.8 (S2), 20.9 (S3), 9.3 (S4); Food waste: 5.2 (S1), 4.6 (S2), 4.6 (S3), 6.5 (S4); Poultry manure: 31.1 (S1), 16.5 (S2), 37.7 (S3), 9.5 (S4).

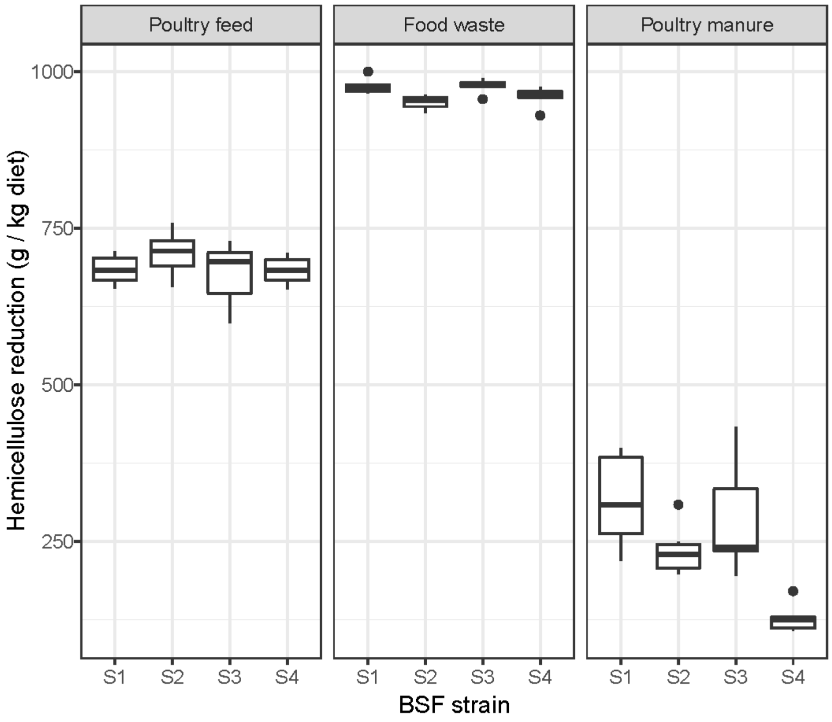

Figure S18. Systemic dietary dry matter losses (emissions).

Boxplots correspond to six replicates within each strain × diet combination. Standard errors of the means (cf. Table 3): Poultry feed: 4.4 (S1), 12.7 (S2), 5.9 (S3), 5.2 (S4); Food waste: 4.6 (S1), 16.7 (S2), 1.4 (S3), 4.5 (S4); Poultry manure: 2.1 (S1), 4.9 (S2), 2.2 (S3), 2.5 (S4).

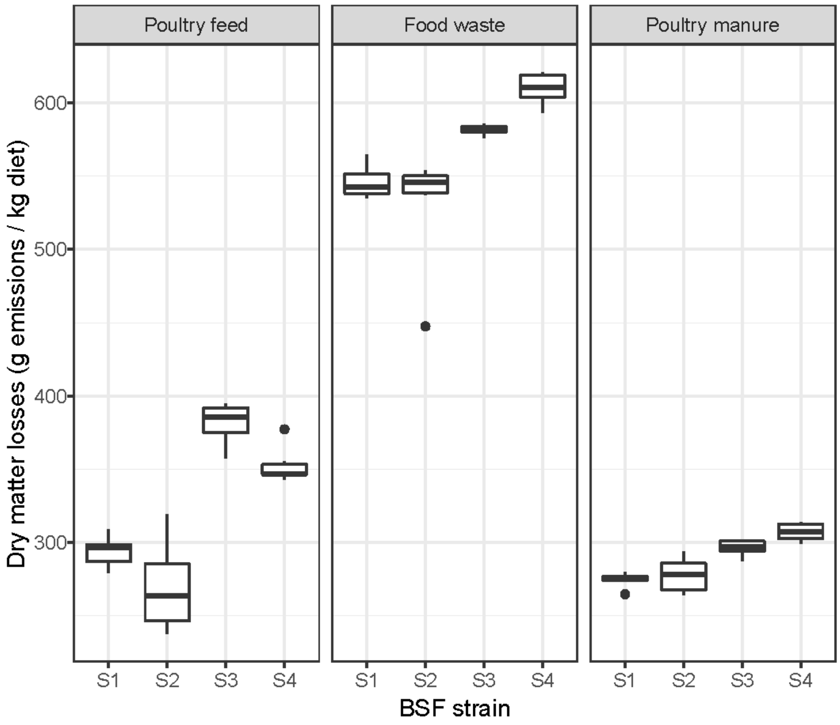

Figure S19. Larval amino acid profiles.

Interaction plots show reaction norms (means ± standard deviations across six replicates per combination) for concentrations (g/100 g BSFL protein; Y-axis) of individual amino acids (abbreviated according to conventions and depicted as alphabetically ordered panels) of four experimental black soldier fly strains (coloured curves) reared on three different diets (X-axis), i.e. poultry feed (PF), food waste (FW) and poultry manure (PM). Asp plus Asn and Glu plus Gln are combined as Asx and Glx, respectively.

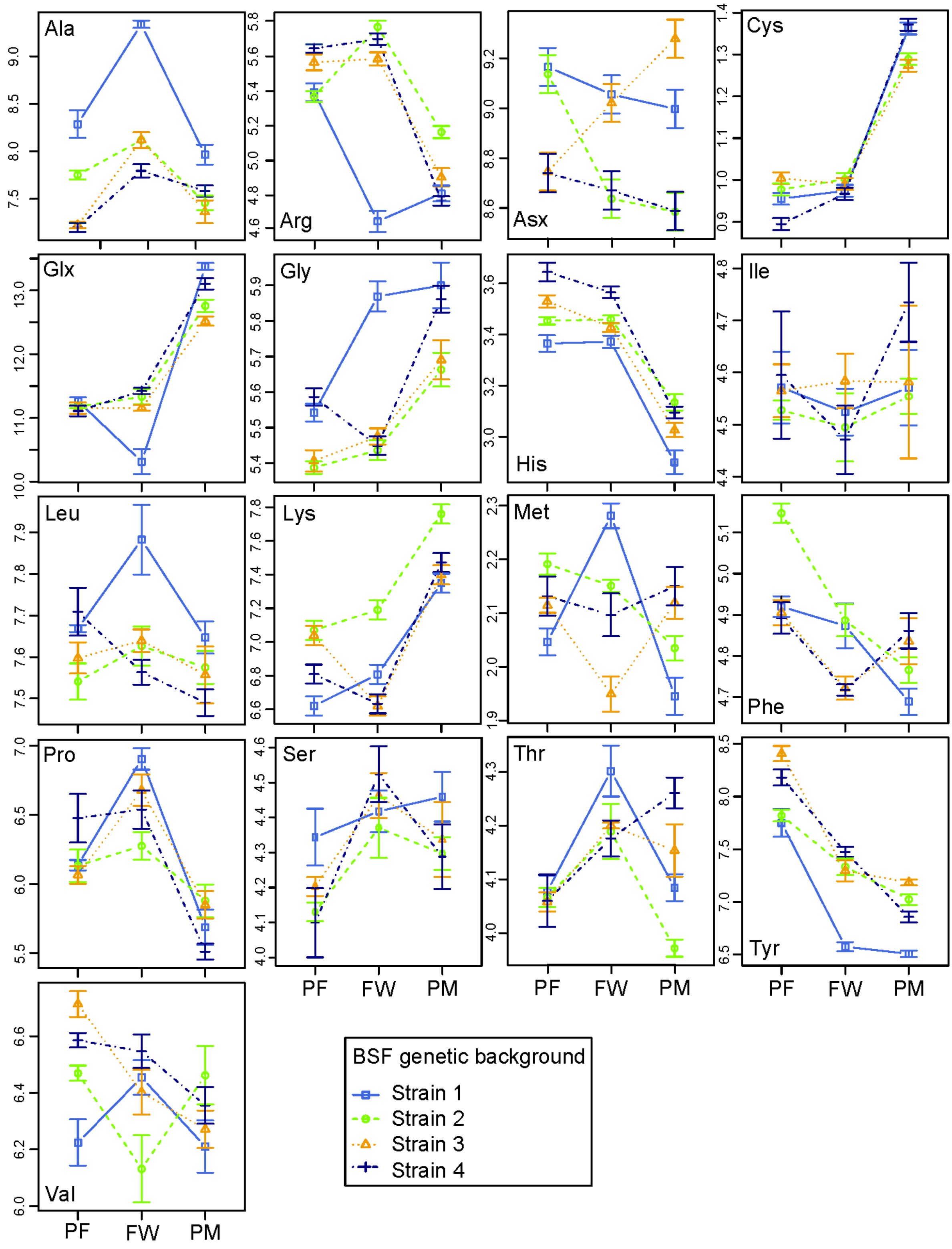

Figure S20. Specific protein concentrations and nitrogen-to-protein conversions.

Interaction plots show reaction norms (means ± standard deviations across six replicates per combination) of four experimental black soldier fly strains (coloured curves) reared on three different diets (X-axes both panels), i.e. poultry feed (PF), food waste (FW) and poultry manure (PM) for A) specific (amino acid based) protein concentrations (dry matter (DM) based; Y-axis) and B) accordingly derived factors for nitrogen-to-protein conversion (*Kp*; Y-axis).

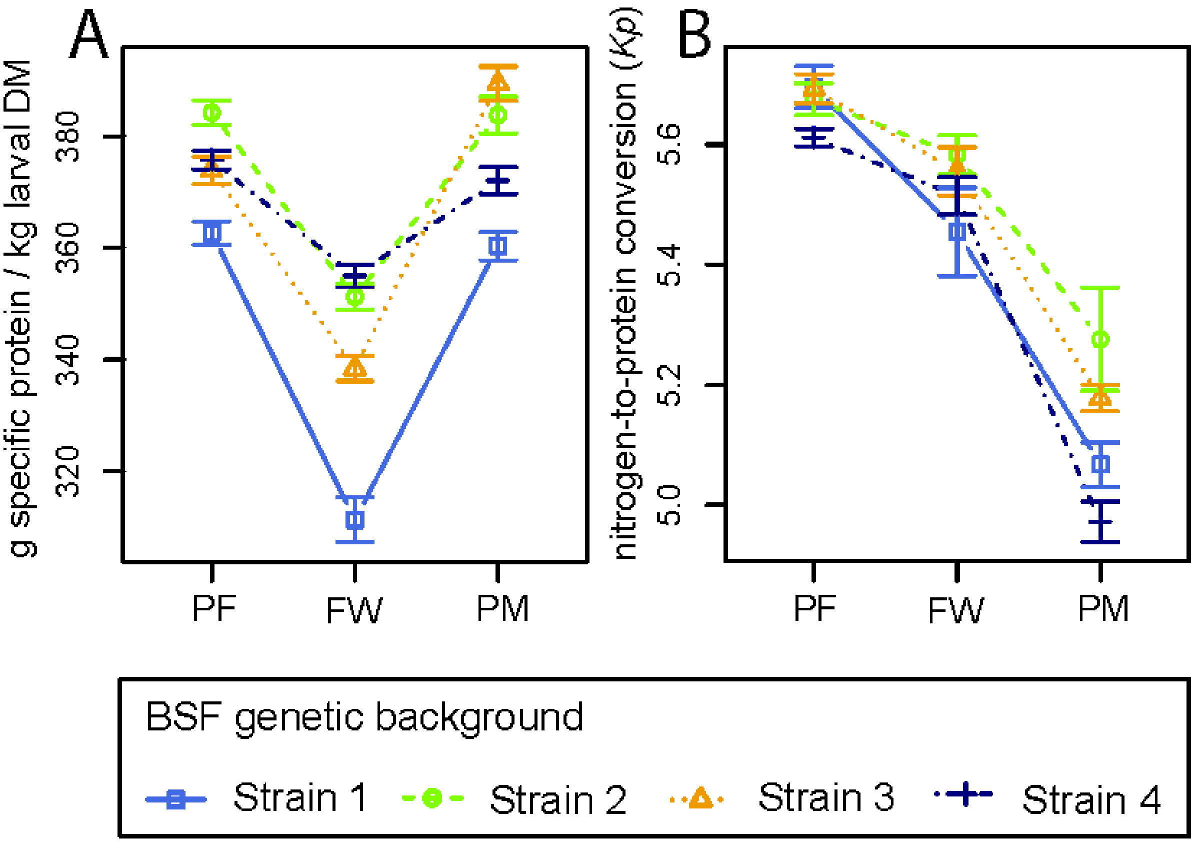

Supplement: Supplementary file 1 [file insects-13-00424-s001.zip › insects-1691970-supplementary.pdf]
